# Competing neural representations of choice shape evidence accumulation in humans

**DOI:** 10.7554/eLife.85223

**Published:** 2023-10-11

**Authors:** Krista Bond, Javier Rasero, Raghav Madan, Jyotika Bahuguna, Jonathan Rubin, Timothy Verstynen

**Affiliations:** 1 Department of Psychology, Carnegie Mellon University Pittsburgh United States; 2 Center for the Neural Basis of Cognition Pittsburgh United States; 3 Carnegie Mellon Neuroscience Institute Pittsburgh United States; 4 Department of Biomedical and Health Informatics, University of Washington Seattle United States; 5 Department of Mathematics, University of Pittsburgh Pittsburgh United States; 6 Department of Biomedical Engineering, Carnegie Mellon University Pittsburgh United States; University Medical Center Hamburg-Eppendorf Germany; Donders Institute for Brain, Cognition and Behaviour Netherlands

**Keywords:** uncertainty, exploration, cortico-basal-ganglia network, Human

## Abstract

Making adaptive choices in dynamic environments requires flexible decision policies. Previously, we showed how shifts in outcome contingency change the evidence accumulation process that determines decision policies. Using in silico experiments to generate predictions, here we show how the cortico-basal ganglia-thalamic (CBGT) circuits can feasibly implement shifts in decision policies. When action contingencies change, dopaminergic plasticity redirects the balance of power, both within and between action representations, to divert the flow of evidence from one option to another. When competition between action representations is highest, the rate of evidence accumulation is the lowest. This prediction was validated in in vivo experiments on human participants, using fMRI, which showed that (1) evoked hemodynamic responses can reliably predict trial-wise choices and (2) competition between action representations, measured using a classifier model, tracked with changes in the rate of evidence accumulation. These results paint a holistic picture of how CBGT circuits manage and adapt the evidence accumulation process in mammals.

## Introduction

Choice is fundamentally driven by information. The process of deciding between available actions is continually updated using incoming sensory signals, processed at a given accumulation rate, until sufficient evidence is reached to trigger one action over another (Gold and Shadlen, 2007; Ratcliff, 1978). The parameters of this evidence accumulation process are highly plastic, adjusting to both the reliability of sensory signals (Nassar et al., 2010; Wilson and Niv, 2011; Nassar et al., 2012; Behrens et al., 2007; Bond et al., 2021) and previous choice history (Urai et al., 2019; Ratcliff and Frank, 2012; Pedersen et al., 2017; Dunovan and Verstynen, 2019; Dunovan et al., 2019; Mendonça et al., 2020), to balance the speed of a given decision with local demands to choose the correct action.

We recently showed how environmental changes influence the decision process by periodically switching the reward associated with a given action in a two-choice task (Bond et al., 2021). This reward contingency change induces competition between old and new action values, leading to a shift in preference toward the new most rewarding option. Internal competition prompts humans to dynamically reduce the rate at which they accumulate evidence (drift rate in a normative drift diffusion model [DDM]; Ratcliff, 1978) and sometimes also increases the threshold of evidence they need to trigger an action (boundary height). The result is a change of the decision policy to a slow, exploratory state. Over time feedback learning pushes the system back into an exploitative state until the environment changes again (see also Dunovan and Verstynen, 2019 and Dunovan et al., 2019).

Here we adopt a generative modeling approach to investigate the underlying neural mechanisms that drive dynamic decision policies in a changing environment. We start with a set of theoretical experiments, using biologically realistic spiking network models, to test how competition within the cortico-basal ganglia-thalamic (CBGT) circuits influences the evidence accumulation process (Dunovan and Verstynen, 2016; Bariselli et al., 2019; Mikhael and Bogacz, 2016; Rubin et al., 2021; Yartsev et al., 2018). Our choice of model, over simple abstracted network models (e.g., rate-based networks), reflects an approach designed to capture both microscale and macroscale dynamics, allowing for the same model to bridge observations across multiple levels of analysis (see also Noble et al., 2020; Schirner et al., 2018; Franklin and Frank, 2015). These theoretical experiments both explain previous results (Bond et al., 2021) and make specific predictions as to how competition between action representations drives changes in the decision policy. We then test these predictions in humans using a high-powered, within-participant neuroimaging design, collecting data over thousands of trials where action–outcome contingencies change on a semi-random basis.

## Results

### CBGT circuits can control decision parameters under uncertainty

**Figure 1.**
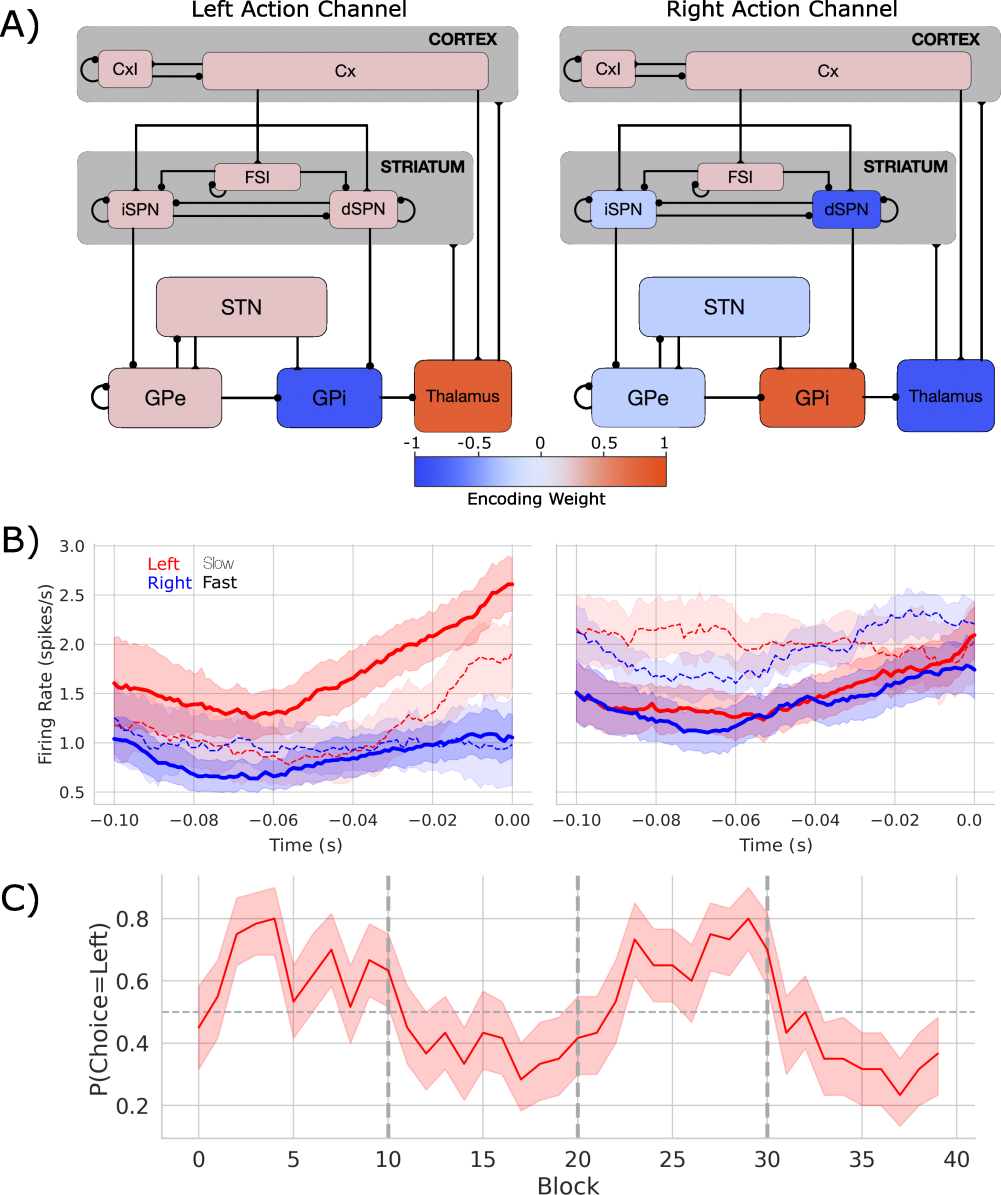
Biologically based cortico-basal ganglia-thalamic (CBGT) network dynamics and behavior. (**A**) Each CBGT nucleus is organized into left and right action channels with the exception of a common population of striatal fast spiking interneurons (FSIs) and cortical interneurons (CxI). Values show encoded weights for left and right action channels when a left action is made. Network schematic adapted from Figure 1 of Vich et al., 2022. (**B**) Firing rate profiles for dSPNs (left panel) and iSPNs (right panel) prior to stimulus onset (t = 0) for a left choice. SPN activity in left and right action channels is shown in red and blue, respectively. Slow and fast decisions are shown with dashed and solid lines, respectively. (**C**) Choice probability for the CBGT network model. The reward for left and right actions changed every 10 trials, marked by vertical dashed lines. The horizontal dashed line represents chance performance.

Both theoretical (Bogacz and Gurney, 2007; Bogacz et al., 2010; Ratcliff and Frank, 2012; Dunovan and Verstynen, 2016; Dunovan et al., 2019; Vich et al., 2022) and experimental (Yartsev et al., 2018; Thura et al., 2022) evidence suggests that the CBGT circuits play a critical role in the evidence accumulation process (for a review, see Gupta et al., 2021). The canonical CBGT circuit (Figure 1A) includes two dissociable control pathways: the direct (facilitation) and indirect (suppression) pathways (Albin et al., 1995; Friend and Kravitz, 2014). A critical assumption of the canonical model is that the basal ganglia are organized into multiple ‘channels,’ mapped to specific action representations (Mink, 1996; Alexander et al., 1986), each containing a direct and indirect pathway. It is important to note that, for the sake of parsimony, we adopt a simple and canonical model of CBGT pathways, with action channels that are agnostic as to the location of representations (e.g., lateralization), simply assuming that actions have unique population-level representations. While a strict, segregated action channel organization may not accurately reflect the true underlying circuitry, striatal neurons have been shown to organize into task-specific spatiotemporal assemblies that qualitatively reflect independent action representations (Adler et al., 2013; Klaus et al., 2017; Barbera et al., 2016; Carrillo-Reid et al., 2011; Badreddine et al., 2022).

**Figure 1—figure supplement 1.**
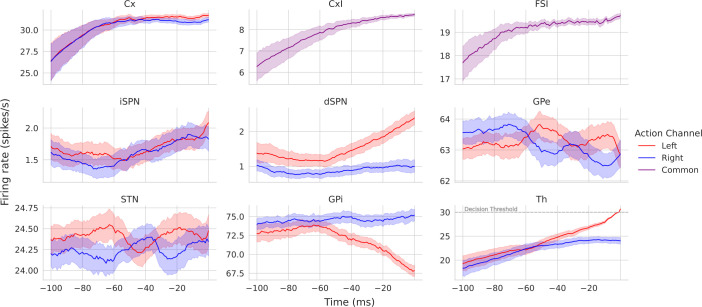
Simulated cortico-basal ganglia-thalamic (CBGT) nuclei firing rates for a left decision. Each panel shows the firing rates for a specific CBGT nucleus starting 100 ms prior to a left decision. The decision threshold for thalamus (30 spikes/s) is marked with a horizontal gray line. Note that the y-axes have different limits for different nuclei due to differences of scale in their firing rates.

**Figure 1—figure supplement 2.**
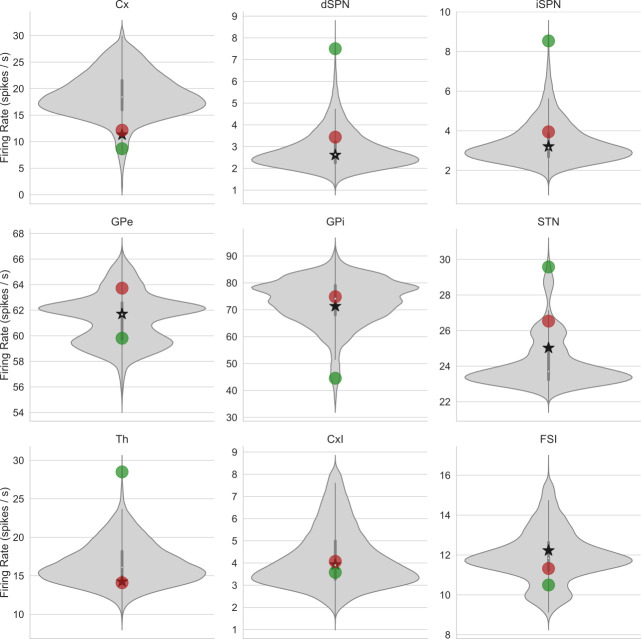
Distributions of cortico-basal ganglia-thalamic (CBGT) nuclei firing rates for slow, intermediate, and fast networks. Each panel shows the distribution of mean firing rates by CBGT nucleus for each of 300 networks sampled. Mean firing rates are shown for each nucleus for slow (red marker), intermediate (black star), and fast (green marker) networks. Again, note the differences of scale in firing rates over nuclei.

**Figure 1—figure supplement 3.**
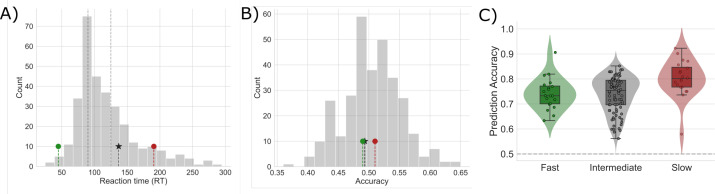
Mean reaction time and accuracy for slow, intermediate, and fast networks. (**A**) The distribution of median reaction times (RTs) for each sampled network. The median RT for sample fast (green marker), intermediate (black star), and slow (red marker) networks are shown. Dashed vertical lines mark the first and second tertiles of the RT distribution. (**B**) The distributions of mean accuracies for each network. (**C**) Prediction accuracy for each type of sampled network. The horizontal dashed line represents chance prediction performance.

**Figure 1—figure supplement 4.**
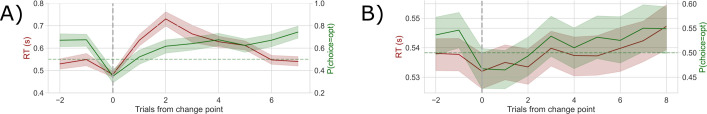
Simulated and human behavior. Change point evoked reaction times are shown in red and accuracy, or the probability of selecting the optimally rewarding choice, is shown in green. Chance is marked as a green horizontal dashed line. The change point is marked by the vertical gray line. (**A**) Simulated behavior. (**B**) Human behavior.

Within these action channels, activation of the direct pathway, via cortical excitation of D1-expressing spiny projection neurons (SPNs) in the striatum, releases GABAergic signals that can suppress activity in the CBGT output nucleus (internal segment of the globus pallidus, GPi, in primates or substantia nigra pars reticulata, SNr, in rodents) (Kravitz et al., 2012; Gurney et al., 2001; Alexander et al., 1986; Mink, 1996). This relieves the thalamus from tonic inhibition, thereby exciting postsynaptic cortical cells and facilitating action execution. Conversely, activation of the indirect pathway via D2-expressing SPNs in the striatum controls firing in the external segment of the globus pallidus (GPe) and the subthalamic nucleus (STN), resulting in strengthened basal ganglia inhibition of the thalamus. This weakens drive to postsynaptic cortical cells and reduces the likelihood that an action is selected in cortex.

Critically, the direct and indirect pathways converge in the GPi/SNr (Kitano et al., 1998; Foster et al., 2021). This suggests that these pathways compete to control whether each specific action is selected (Dunovan et al., 2015). The apparent winner-take-all selection policy and action-channel like coding (Adler et al., 2013; Klaus et al., 2017; Barbera et al., 2016; Carrillo-Reid et al., 2011; Badreddine et al., 2022) also imply that action representations themselves compete. Altogether, this neuroanatomical evidence suggests that competition both between and within CBGT pathways control the rate of evidence accumulation during decision-making (Dunovan et al., 2019; Bariselli et al., 2019; Vich et al., 2022).

To simulate this process, we designed a spiking neural network model of the CBGT circuits, shown in Figure 1A, with dopamine-dependent plasticity occurring at the corticostriatal synapses (Vich et al., 2020; Rubin et al., 2021). Critically, although this model simulates dynamics that happen on a microscale, it can be mapped upward to infer macroscale properties, like inter-region dynamics and complex behavior, making it a useful theoretical tool for bridging across levels of analysis. The network performed a probabilistic two-arm bandit task with switching reward contingencies (see Figure 3—figure supplement 1) that followed the same general structure as our prior work (Bond et al., 2021), with the exception that block switches were deterministic for the model, happening every 10 trials, whereas in actual experiments they are generated probabilistically so as to increase the uncertainty of participant expectations of the timing of outcome switches. In brief, the network selected one of two targets, each of which returned a reward according to a specific probability distribution. The relative reward probabilities for each target were held constant at 75 and 25% and the action–outcome contingency was changed every 10 trials, on average. For the purpose of this study, we focus primarily on the neural and behavioral effects associated with a switch in the identity of the optimal target. We used four different network instances (see Supp. Methods) as a proxy for simulating individual differences over human participants.

Figure 1B shows the firing rates of dSPNs and iSPNs in the left action channel, time-locked to selection onset (when thalamic units exceed 30 Hz, t = 0), for both fast (<196 ms) and slow (>314.5 ms) decisions (see Figure 1—figure supplement 1 for node-by-node firing-rates). As expected, the dSPNs show a ramping of activity as decision onset is approached and the slope of this ramp scales with response speed. In contrast, we see that iSPN firing is sustained during slow movements and weakly ramps during fast movements. However, iSPN firing was relatively insensitive to left versus right decisions. This is consistent with our previous work showing that differences in direct pathways track primarily with choice while indirect pathway activity modulates overall response speeds (Dunovan et al., 2019; Vich et al., 2022) as supported by experimental studies (Yttri and Dudman, 2016; Forstmann et al., 2010; Maia and Frank, 2011).

**Figure 2.**
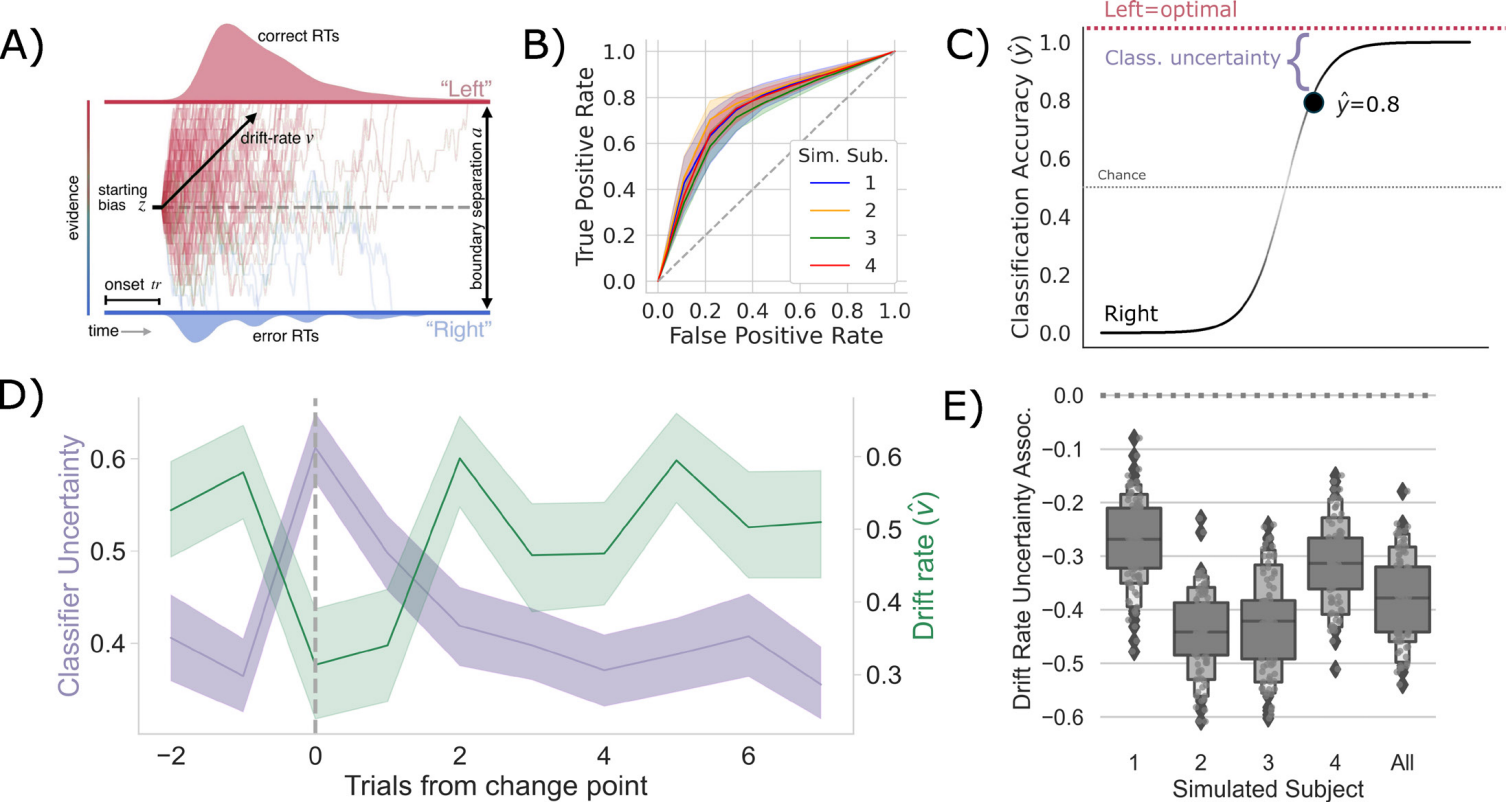
Competition between action plans *should* drive evidence accumulation. (**A**) Decision parameters were estimated by modeling the joint distribution of reaction times and responses within a drift diffusion framework. (**B**) Classification performance for single-trial left and right actions shown as an Receiver Operating Characteristic (ROC) curve. The gray dashed line represents chance performance. (**C**) Predicted left and right responses. The distance of the predicted response from the optimal choice represents uncertainty for each trial. For example, here the predicted probability of a left response on the first trial ${\hat{y}}_{t_{1}}$ is 0.8. The distance from the optimal choice on this trial and, thereby, the uncertainty $u_{t_{1}}$, is 0.2. (**D**) Change point-evoked uncertainty (lavender) and drift rate (green). The change point is marked by a dashed line. (**E**) Bootstrapped estimates of the association between uncertainty and drift rate. Results for individual participants are presented along with aggregated results.

We then modeled the behavior of the CBGT network using a hierarchical version of the DDM (Wiecki et al., 2013), a canonical formalism for the process of evidence accumulation during decision-making (Ratcliff, 1978; Figure 2A). This model returns four key parameters with distinct influences on evidence accumulation. The drift rate (v) represents the rate of evidence accumulation, the boundary height (a) represents the amount of evidence required to cross the decision threshold, nondecision time (t) is the delay in the onset of the accumulation process, and starting bias (z) is a bias to begin accumulating evidence for one choice over another (see ‘Materials and methods’ section).

**Figure 2—figure supplement 1.**
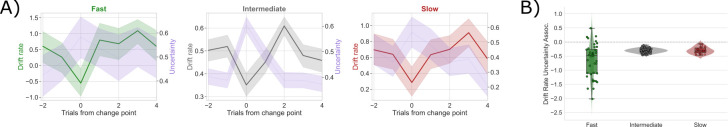
Competition between action plans drives evidence accumulation for fast, intermediate, and slow networks. (**A**) Classifier uncertainty (lavender) and estimated drift rate (fast: green, intermediate: gray, and slow: red) dynamics. (**B**) Bootstrapped estimates of the association between classifier uncertainty and drift rate for each type of network.

We tracked internal estimates of action-value and environmental change using trial-by-trial estimates of two ideal observer parameters, the belief in the value of the optimal choice (Δ⁢B) and change point probability (Ω), respectively (see Bond et al., 2021; Nassar et al., 2010 and ‘Materials and methods’ for details). Using these estimates, we evaluated how a suspected change in the environment and the belief in optimal choice value influenced underlying decision parameters. Consistent with prior observations in humans (Bond et al., 2021), we found that both v and a were the most pliable parameters across experimental conditions for the network. Specifically, we found that the model mapping Δ⁢B to drift rate and Ω to boundary height and the model relating Δ⁢B to drift rate provided equivocal best fits to the data over human participants ($\Delta \,D\,I\,C_{\text{null}}=-29.85\pm 12.76$ and $\Delta \,D\,I\,C_{\text{null}}=-22.60\pm 7.28$, respectively; see Burnham and Anderson, 1998 and ‘Materials and methods’ for guidelines on model fit interpretation). All other models failed to provide a better fit than the null model (Supplementary file 1). Consistent with prior work (Bond et al., 2021), we found that the relationship between Ω and the boundary height was unreliable (mean $\beta_{a∼\Omega }=0.069\pm 0.152$; mean p=0.232±0.366). However, drift rate reliably increased with Δ⁢B in three of four participants (mean $\beta_{v∼\Delta \,B}=0.934\pm 0.386$; mean p<0.001 participants p<0.001; Supplementary file 2).

These effects reflect a stereotyped trajectory around a change point, whereby v immediately plummets and a briefly increases, with a quickly recovering and v slowly growing as reward feedback reinforces the new optimal target (Bond et al., 2021). Because prior work has shown that the change in v is more reliable than changes in a (Bond et al., 2021) and because v determines the direction of choice, we focus the remainder of our analysis on the control of v.

To test whether these shifts in v are driven by competition within and between action channels, we predicted the network’s decision on each trial using a LASSO-PCR trained on the pre-decision firing rates of the network (see ‘Measuring neural action representations’). The choice of LASSO-PCR was based on prior work building reliable classifiers from whole-brain-evoked responses that maximize inferential utility (see Meissner et al., 2011). The method is used when models are over-parameterized, as when there are more voxels than observations, relying on a combination of dimensionality reduction and sparsity constraints to find the true, effective complexity of a given model. While these are not considerations with our network model, they are with the human validation experiment that we describe next. Thus, we used the same classifier on our model as on our human participants to directly compare theoretical predictions and empirical observations. Model performance was cross-validated at the run level using a leave-one-run-out procedure, resulting in 45 folds per subject (five runs for each of the nine sessions). We then classified all trials in the hold-out set to evaluate prediction accuracy. The cross-validated accuracy for the four models, simulating individual participants, is shown in Figure 2B as ROC curves. The classifier was able to predict the chosen action with approximately 75% accuracy (72–80%) for each simulated participant, with an average area under the curve (AUC) of approximately 0.75, ranging from 0.71 to 0.77.

Examining the encoding pattern in the simulated network, we see lateralized activation over left and right action channels (Figure 1A), with opposing weights in GPi and thalamus, and, to a lesser degree, contralateral encoding in STN and in both indirect and direct SPNs in striatum. We do not observe contralateral encoding in cortex, which likely reflects the emphasis on basal ganglia structures and lumped representation of cortex in the model design.

To quantify the competition between action channels, we took the unthresholded prediction from the LASSO-PCR classifier, ${\hat{y}}_{t}$, and calculated its distance from the optimal target (i.e., target with the highest reward probability) on each trial (Figure 2C). This provided an estimate of the uncertainty driven by the separability of pre-decision activity across action channels. In other words, the distance from the optimal target should increase with increased co-activation of circuits that represent opposing actions. The decision to model aggregate trial dynamics with a classifier stems from the limitations of the hemodynamic response that we will use next to vet the model predictions in humans. The low temporal resolution of the evoked BOLD signal makes finer-grained temporal analysis for the human data impossible as the signal is a low-pass filtered version of the aggregate response over the entire trial. So, we chose to represent the macroscopic network dynamics as classifier uncertainty, which cleanly links the cognitive model results to both behavior and neural dynamics at the trial-by-trial level using only two variables (drift rate and classifier uncertainty). This approach allows us to directly compare model and human results.

If the competition in action channels is also driving v, then there should be a negative correlation between the classifier’s uncertainty and v, particularly around a change point. Indeed, this is exactly what we see (Figure 2D). In fact, the uncertainty and v are consistently negatively correlated across all trials in every simulated participant and in aggregate (Figure 2E). Thus, in our model of the CBGT pathways, competition between action representations drives changes in v in response to environmental change.

Next, in order to rule out the possibility that these adaptive network effects emerged due to the specific parameter scheme that we used for the simulations, we re-ran our simulations using different parameter schemes. For this, we used a constrained sampling procedure (see Vich et al., 2022) to sample a range of different networks with varying degrees of speed and accuracy. This parameter search was constrained to permit regimes that result in biologically realistic firing rates (Figure 1—figure supplement 1). The simulations above arose from a parameter scheme lying in the middle of this response speed distribution (intermediate). We then chose two parameter regimes, one that produces response speeds in the upper quartile of the distribution (slow) and one that produces response speeds in the lower quartile (fast; Figure 1—figure supplement 2A and B). We repeated the simulation experiments with these new more ‘extreme’ networks. As expected, our general classifier accuracy held across the range of regimes, with comparable performance across all three model types (Figure 1—figure supplement 2C). In addition, the reciprocal relationship between classifier uncertainty and v were replicated in the fast and slow networks (Figure 2—figure supplement 1A), with the fast network showing a more expansive dynamic range of drift rates than the intermediate or slow networks. When we look at the correlation between classifier uncertainty and v, we again see a consistent negative association across parameter regimes (Figure 2—figure supplement 1B). The variability of this effect increases when networks have faster response times, suggesting that certain parameter regimes increase overall behavioral variability. Despite this, our key simulation effects appear to be robust to variation in parameter scheme.

### Humans adapt decision policies in response to change

To test the predictions of our model, a sample of humans (N = 4) played a dynamic two-armed bandit task under experimental conditions similar to those used for the simulated CBGT network and prior behavioral work (Bond et al., 2021) as whole brain hemophysiological signals were recorded using functional magnetic resonance imaging (fMRI) (Figure 3—figure supplement 1). On each trial, participants were presented with a male and female Greeble (Gauthier and Tarr, 1997). The goal was to select the Greeble most likely to give a reward. Selections were made by pressing a button with their left or right hand to indicate the left or right Greeble on the screen.

Crucially, we designed this experiment such that each participant acted as an out-of-set replication test, having performed thousands of trials individually. Specifically, to ensure we had the statistical power to detect effects on a participant-by-participant basis, we collected an extensive data set comprising 2700 trials over 45 runs from nine separate imaging sessions for each of four participants. Consequently, we amassed a grand total of 36 hr of imaging data over all participants, which was used to evaluate the replicability of our findings at the participant-by-participant level. Therefore, our statistical analyses were able to estimate effects on a single-participant basis.

Behaviorally, human performance in the task replicated our prior work (Bond et al., 2021). Both response speed and accuracy changed across conditions in a way that matched what we observed in Experiment 2 in Bond et al., 2021. Specifically, we see a consistent effect of change point on both RT and accuracy that matches the behavior of our network (Figure 3—figure supplement 2). To address how a change in the environment shifted underlying decision dynamics, we used a hierarchical DDM modeling approach (Wiecki et al., 2013) as we did with the network behavior (see ‘Materials and methods’ for details). Given previous empirical work (Bond et al., 2021) and the results from our CBGT network model showing that only v and, less reliably, a respond to a shift in the environment, we focused our subsequent analysis on these two parameters. We compared models where single parameters changed in response to a switch, pairwise models where both parameters changed, and a null model that predicts no change in decision policy (Supplementary files 1 and 2). Consistent with the predictions from our CBGT model, we found equivocal fits for the model mapping both Δ⁢B to v and Ω to a and a simpler model mapping Δ⁢B to v (see Supplementary file 1 for average results). This pattern was fairly consistent at the participant level, with 3/4 participants showing Δ⁢B modulating v (Supplementary file 2). These results suggest that as the belief in the value of the optimal choice approaches the reward value for the optimal choice, the rate of evidence accumulation increases.

Taken altogether, we confirm that humans rapidly shift how quickly they accumulate evidence (and, to some degree, how much evidence they need to make a decision) in response to a change in action–outcome contingencies. This mirrors the decision parameter dynamics predicted by the CBGT model. We next evaluated how this change in decision policy tracks with competition in neural action representations.

### Measuring action representations in the brain

To measure competition in action representations, we first needed to determine how individual regions (i.e., voxels) contribute to single decisions. For each participant, trial-wise responses at every voxel were estimated by means of a general linear model (GLM), with trial modeled as a separate condition in the design matrix. Therefore, the ${\hat{\beta }}_{t,v}$ estimated at voxel v reflected the magnitude of the evoked response on trial t. As in the CBGT model analysis, these whole-brain, single-trial responses were then submitted to a LASSO-PCR classifier to predict left/right response choices (Figure 3—figure supplement 3). The performance of the classifier for each participant was evaluated with a 45-fold cross-validation, iterating through all runs so that each one corresponded to the hold-out test set for one fold.

**Figure 3.**
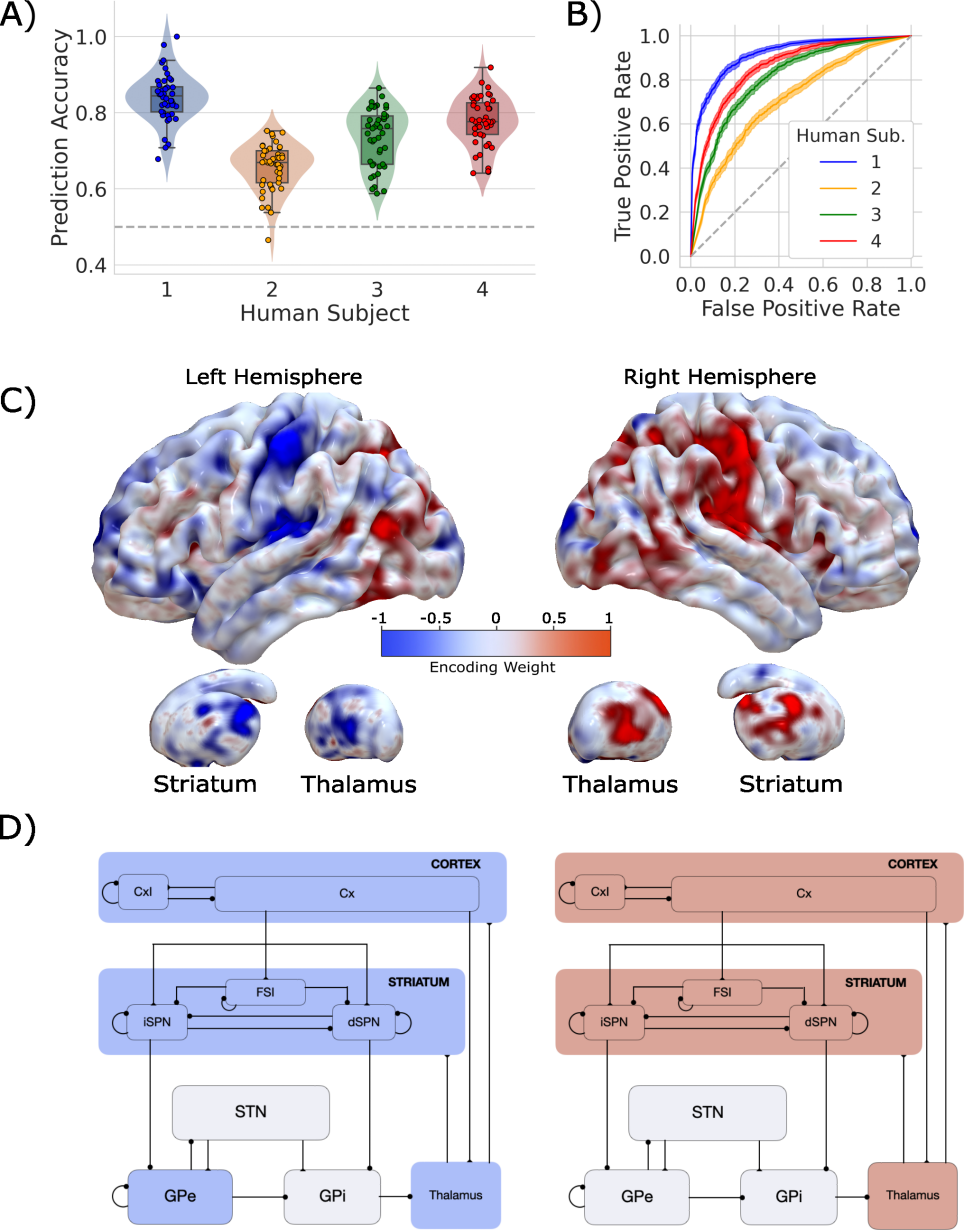
Single-trial prediction of action plan competition in humans. (**A**) Overall classification accuracy for single-trial actions for each participant. Each point corresponds to the performance for each of the 45 folds in our leave-one-run-out cross-validation procedure. (**B**) Classification performance for single-trial actions shown as an ROC curve. The gray dashed line represents chance performance. (**C**) Participant-averaged encoding weight maps in standard space for both hemispheres. (**D**) The mean encoding weights within each cortico-basal ganglia-thalamic (CBGT) node in both hemispheres. See encoding weight scale above for reference.

Our classifier was able to predict single-trial responses well above chance for each of the four participants (Figure 3A and B), with mean prediction accuracy ranging from 65 to 83% (AUCs from 0.72 to 0.92). Thus, as with the CBGT network model, we were able to reliably predict trial-wise responses for each participant. Figure 3C shows the average encoding map for our model as an illustration of the influence of each voxel on our model predictions (Figure 3—figure supplement 4 displays individual participant maps). These maps effectively show voxel-tuning toward rightward (blue) or leftward (red) responses. Qualitatively, we see that cortex, striatum, and thalamus all exhibit strongly lateralized influences on contralateral response prediction. Indeed, when we average the encoding weights in terms of principal CBGT nuclei (Figure 3D), we confirm that these three regions largely predict contralateral responses. See Figure 3—figure supplement 4 for a more detailed summary of the encoding weights across multiple cortical and subcortical regions.

**Figure 3—figure supplement 1.**
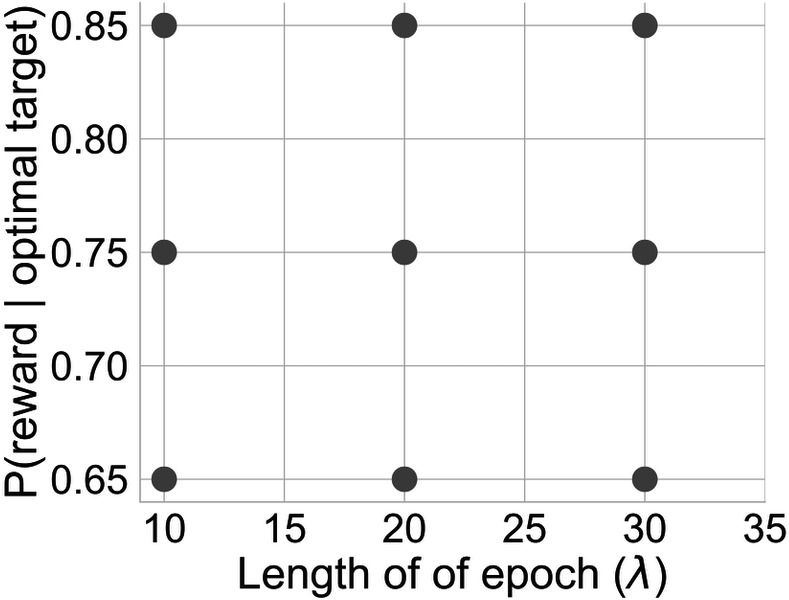
Experimental design. The probability of reward for the statistically optimal target (conflict; y-axis) and the rate at which the optimal target shifted (volatility; x-axis) were manipulated according to this design, with each point representing a combination of the two variables. High conflict resulted in a smaller difference in the probability of reward between the optimal and suboptimal targets, while high volatility resulted in frequent switches in the optimal target selection.

**Figure 3—figure supplement 2.**
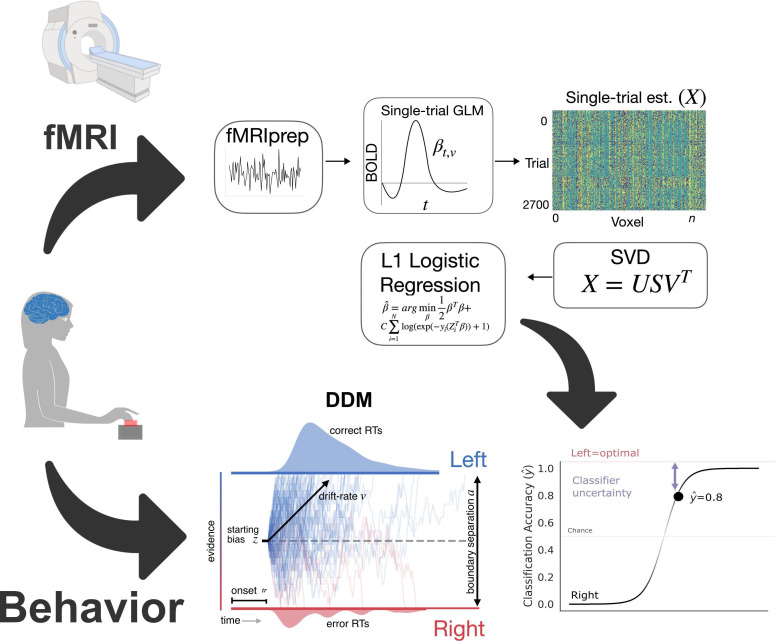
Analysis method. *Inputs*. Behavioral responses in the form of reaction time and accuracy, along with trial-by-trial hemodynamic responses, were collected as participants learned the task. In the case of the simulated cortico-basal ganglia-thalamic (CBGT) network, this step involved simulating responses to experimental manipulations. *Preprocessing*. Data underwent standard preprocessing and then we estimated single-trial hemodynamic responses. *Cross-validated prediction model*. We reduced the dimensionality of the trial estimate matrix, X, using singular-value decomposition. Then we conducted logistic regression with a sparsity penalty (L1-norm). *Outputs*. For the imaging data, we predicted left or right responses, here coded as 0 or 1. We calculated classifier uncertainty from the unthresholded response prediction. The distance of this predicted response from the optimal choice represents classifier uncertainty for each trial. Here, the predicted probability of a left response is 0.8. The distance from the optimal choice on this trial, and, thereby, the classifier uncertainty is 0.2. The joint distribution of reaction times and accuracies were also fitted to estimate latent decision parameters using the drift diffusion modelDrift Diffusion Model (DDM).

**Figure 3—figure supplement 3.**
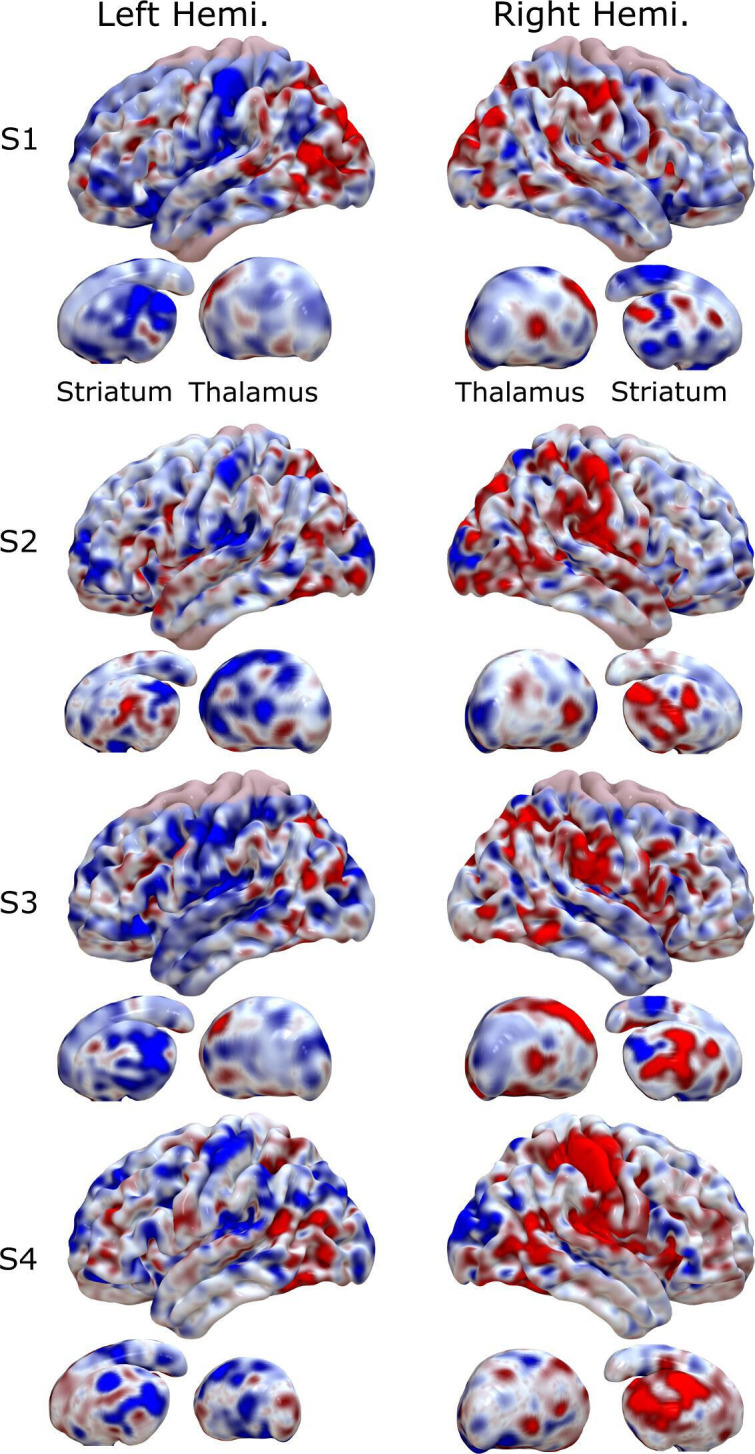
Encoding maps in standardized space for each participant. Rows represent individual participants. Columns refer to left and right views of the whole brain. Thalamus and striatum are shown beneath each cortical map. Values are z-scored.

**Figure 3—figure supplement 4.**
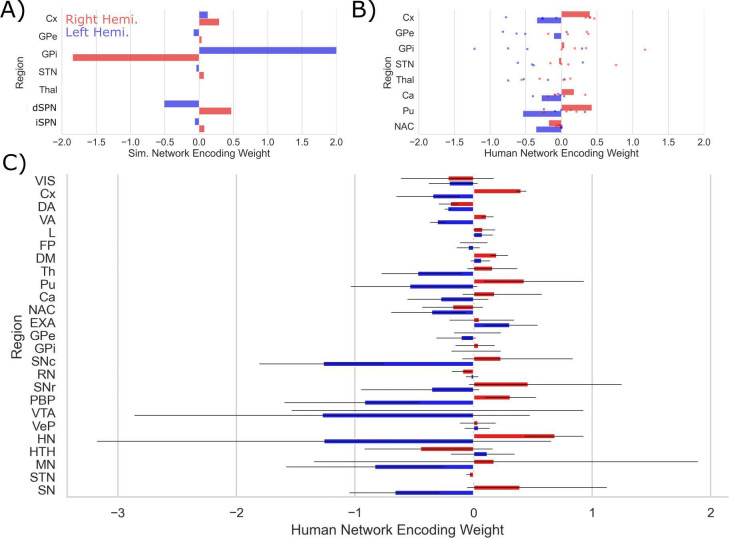
Encoding patterns by CBGT node. (**A**) Simulated CBGT encoding weights. (**B**) Human CBGT encoding weights for comparison with the simulated CBGT network results. Each point represents the average result for each participant. Bars represent participant-averaged data. (**C**) The full set of human CBGT encoding weights for all captured nodes from whole-brain imaging. Gray error bars represent 95% CIs over participants. Left hemisphere weights are marked in blue and right hemisphere weights are marked in red.

These results show that we can reliably predict single-trial choices from whole-brain hemodynamic responses for individual participants. Further, key regions of the CBGT pathway contribute to these predictions. Next, we set out to determine whether competition between these representations for left and right actions correlates with changes in the drift rate, as predicted by the CBGT network model (Figure 2C).

### Competition between action representations may drive drift rate

To evaluate whether competition between action channels correlates with the magnitude of v on each trial, as the CBGT network predicts (Figure 2C), we focused our analysis on trials surrounding the change point, following analytical methods identical to those described in the previous section and shown in Figure 2C.

**Figure 4.**
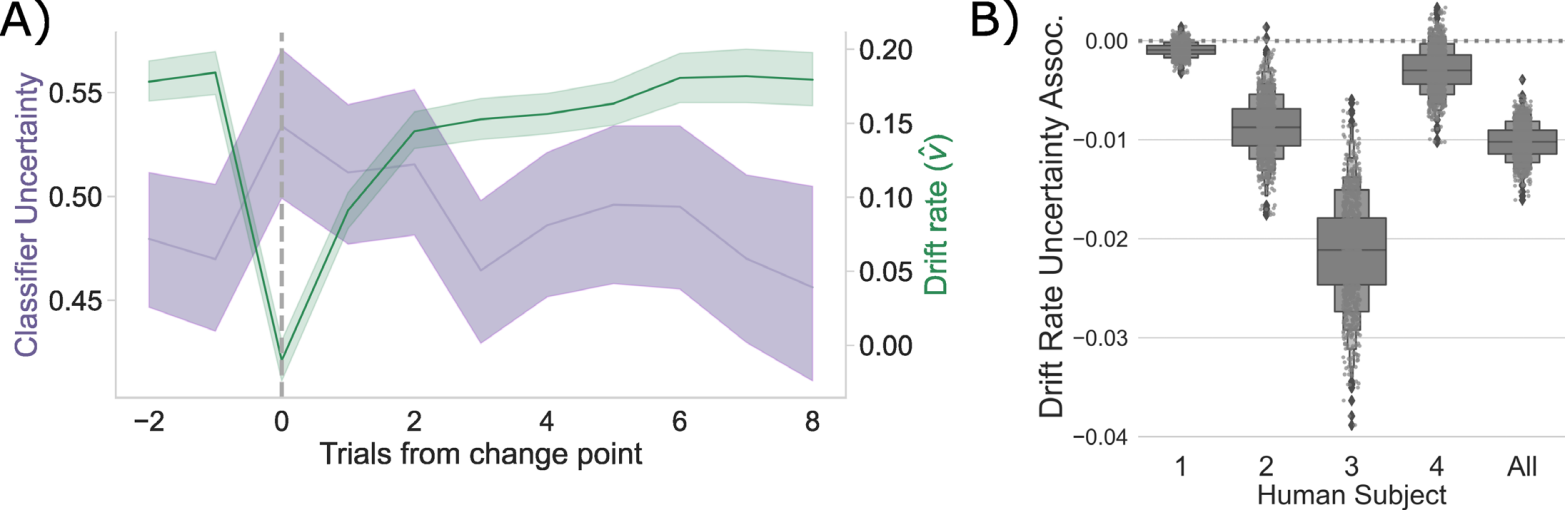
Competition between action plans drives evidence accumulation in humans. (**A**) Classifier uncertainty (lavender) and estimated drift rate ($\hat{v}$; green) dynamics. (**B**) Bootstrapped estimate of the association between classifier uncertainty and drift rate by participant and in aggregate.

Consistent with the CBGT network model predictions, following a change point, v shows a stereotyped drop and recovery as observed in the CBGT network (Figure 2C) and prior behavioral work (Bond et al., 2021; Figure 4A). This drop in v tracked with a relative increase in classifier uncertainty, and subsequent recovery, in response to a change in action–outcome contingencies (mean bootstrapped β:−0.021 to −0.001; t range: -3.996 to $-1.326;\;ps_{1}=0.057$, $p_{S2}<0.001$, $p_{\text{All}}<0.001$). As with the CBGT network simulations (Figure 2D), we also observe a consistent negative correlation between v and classifier uncertainty over all trials, irrespective of their position to a change point, in each participant and in aggregate (Figure 4B; Spearman’s ρ range: -0.08 to −0.04; p range: <0.001 to 0.043, see Figure 4—figure supplement 1 for null effect on a).

**Figure 4—figure supplement 1.**
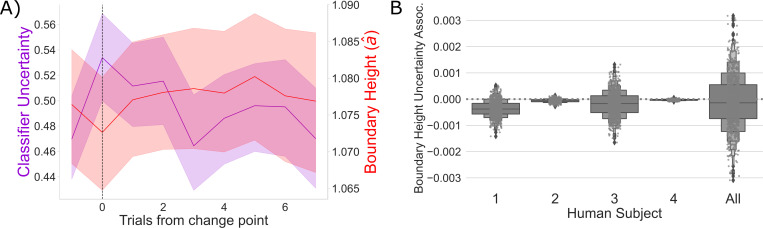
Competition between action plans and boundary height. (**A**) Change point-evoked classifier uncertainty (lavender) and estimated boundary height (red). Bootstrapped 95% CIs are shown. (**B**) The association between classifier uncertainty and boundary height by participant and in aggregate.

These results clearly suggest that, as predicted by our CBGT network simulations and prior work (Dunovan et al., 2019; Vich et al., 2022; Rubin et al., 2021), competition between action representations drives changes in the rate of evidence accumulation during decision-making in humans.

## Discussion

Here we investigated the underlying mechanisms that drive shifts in decision policies when the rules of the environment change. We first tested an implementation-level theory of how CBGT networks contribute to changes in decision policy parameters using a modeling approach that allows us to bridge across levels of analysis. This theory predicted that the rate of evidence accumulation is driven by competition across action representations. Using a high-powered, within-participants fMRI design, where each participant served as an independent replication test, we found evidence consistent with our CBGT network simulations. Specifically, as action–outcome contingencies change, thereby increasing uncertainty in the optimal choice, decision policies shift with a rapid decrease in the rate of evidence accumulation, followed by a gradual recovery to baseline rates as new contingencies are learned (see also Bond et al., 2021). These results empirically validate prior theoretical and computational work predicting that competition between neural populations encoding distinct actions modulates how information is used to drive a decision (Bogacz and Gurney, 2007; Bogacz et al., 2010; Ratcliff and Frank, 2012; Dunovan and Verstynen, 2016; Dunovan et al., 2019).

Our findings here align with prior work on the role of competition in the regulation of evidence accumulation. In the decision-making context, the ratio of dSPN to iSPN activation *within* an action channel has been linked to the drift rate of single-action decisions (Dunovan et al., 2015; Dunovan and Verstynen, 2016; Bariselli et al., 2019; Mikhael and Bogacz, 2016). In the motor control context, this competition manifests as movement vigor (Yttri and Dudman, 2016; Dudman and Krakauer, 2016; Turner and Desmurget, 2010). Yet, our results show how competition *across* channels drives drift rate dynamics. So how do we reconcile these two effects? Mechanistically, the strength of each action channel is defined by the relative difference between dSPN and iSPN influence. In this way, competition across action channels is defined by the relative balance of direct and indirect pathway activation within each channel. Greater within-channel competition, relative to the competition in other channels, makes that action decision relatively slow and reduces the overall likelihood that it is selected. This mechanism is consistent with prior theoretical (Dunovan et al., 2019; Vich et al., 2022) and empirical work (Yartsev et al., 2018).

While our current work postulates a mechanism by which changes in action–outcome contingencies drive changes in evidence accumulation through plasticity within the CBGT circuits, the results presented here are far from conclusive. For example, our model of the underlying neural dynamics predicts that the certainty of individual action representations is encoded by the competition between direct and indirect pathways (see also Dunovan et al., 2019; Vich et al., 2020; Vich et al., 2022). Thus, external perturbation of dSPN (or iSPN) firing during decision-making, say using optogenetic stimulation, should causally impact the evidence accumulation rate and, subsequently, the speed at which the new action–outcome contingencies are learned. Indeed, there is already some evidence for this outcome (see Yartsev et al., 2018, but also Ding and Gold, 2010 for contrastive evidence).

Our model, however, has very specific predictions with regards to disruptions of each pathway within an action representation. Disrupting the *balance* of dSPN and iSPN efficacy should selectively impact the drift rate (and, to a degree, onset bias; see Vich et al., 2022), while non-specific disruption of global iSPN efficacy across action representations should selectively disrupt boundary height (and, to a degree, accumulation onset time; see again Vich et al., 2022). These are specific predictions that can be tested in follow-up studies.

Careful attention to the effect size of our correlations between channel competition and drift rate shows that the effect is substantially smaller in humans than in the model. This is not surprising due to several factors. Firstly, the simulated data is not affected by the same sources of noise as the hemodynamic signal, whose responses can be greatly influenced by factors such as the heterogeneity of cell populations and the properties of underlying neurovascular coupling. Additionally, our model is not susceptible to non-task-related variance, such as fatigue or lapses of attention, which the humans likely experienced. While we could have fine-tuned the model results based on the empirical human data, that would contaminate the independence of our predictions and defeat the purpose of using a generative model. With this in mind, we opted to focus on comparing the overall pattern of human and simulated results. Finally, our simulations only used a single experimental condition, whereas human experiments varied the relative value of options and the volatility of their value, which led to more variance in human responses. Nevertheless, despite these differences, we see qualitative similarities in both the model and human results, providing confirmation of a key aspect of our theory.

Looking at the overall pattern of results, we see that increasing the difference between dSPN and iSPN firing in the channel representing the new optimal-action should decrease the time needed to resolve the credit assignment problem during learning (Rubin et al., 2021). This would result in faster and more accurate learning in response to a change in the environment and lead to characteristic signatures in the distribution of reaction times, as well as choice probabilities, reflective of a shift in evidence accumulation rate. Of course, testing these predictions is left to future work.

It is important to point out that there are critical assumptions in our model that might impact how the results can be interpreted. For example, we are assuming a strict action channel organization of CBGT pathways (Mink, 1996). Realistically action representations in these networks are not as rigid, and there may be overlaps in these representations (see Klaus et al., 2017). However, by restricting our responses to fingers on opposite hands, it is reasonable to assume that the underlying CBGT networks that regulate selecting the two actions are largely independent. Another critical assumption of our model is the simple gating mechanism from the thalamus, where actions get triggered once thalamic firing crosses a specified threshold. In reality, the dynamics of thalamic gating are likely more complicated (Logiaco et al., 2021) and the nuance of this process could impact network behavior and subsequent predictions. Until the field has a better understanding of the process of gating actions, our simple threshold model, although incomplete, remains useful for generating simple behavioral predictions. These assumptions may limit some of the nuance of the predicted brain–behavior associations; however, they likely have little impact on the main prediction that competition in action representations tracks with the rate of evidence accumulation during decision-making.

### Conclusion

As the world changes and certain actions become less optimal, successful behavioral adaptation requires flexibly changing how sensory evidence drives decisions. Our simulations and hemophysiological experiments in humans show how this process can occur within the CBGT circuits. Here, a shift in action–outcome contingencies induces competition between encoded action plans by modifying the relative balance of direct and indirect pathway activity in CBGT circuits, both within and between action channels, slowing the rate of evidence accumulation to promote adaptive exploration.

If the environment subsequently remains stable, then this learning process accelerates the rate of evidence accumulation for the optimal decision by increasing the strength of action representations for the new optimal choice. This highlights how these macroscopic systems promote flexible, effective decision-making under dynamic environmental conditions.

## Materials and methods

### Simulations

We simulated neural dynamics and behavior using a biologically based, spiking CBGT network model (Dunovan and Verstynen, 2019; Vich et al., 2022). The network representing the CBGT circuit is composed of nine neural populations: cortical interneurons (CxI), excitatory cortical neurons (Cx), striatal D1/D2-spiny projection neurons (dSPNs/iSPNs), striatal fast-spiking interneurons (FSI), the internal (GPi) and external globus pallidus (GPe), the subthalamic nucleus (STN), and the thalamus (Th). All the neuronal populations are segregated into two action channels with the exception of cortical (CxI) and striatal interneurons (FSIs). Each neuron in the population was modeled with an integrate-fire-or-burst-model (Wei et al., 2015), and a conductance-based synapse model was used for NMDA, AMPA, and GABA receptors. The neuronal and network parameters (inter-nuclei connectivity and synaptic strengths) were tuned to obtain realistic baseline firing rates for all the nuclei. The details of the model are described in our previous work (Vich et al., 2022) as well as in the ‘Neuron model’ section below for the sake of completeness.

Corticostriatal weights for D1 and D2 neurons in striatum were modulated by phasic dopamine to model the influence of reinforcement learning on network dynamics. The details of STDP learning are described in detail in previous work (Vich et al., 2020), but key details are shown below. As a result of these features of the CBGT network, it was capable of learning under realistic experimental paradigms with probabilistic reinforcement schemes (i.e., under reward probabilities and unstable action–outcome values).

### Threshold for CBGT network decisions

A decision between the two competing actions (‘left’ and ‘right’) was considered to be made when either of the thalamic subpopulations reached a threshold of 30 Hz. This threshold was set based on the network dynamics for the chosen parameters with the aim of obtaining realistic reaction times. The maximum time allowed to reach a decision was 1000 ms. If none of the thalamic subpopulations reached the threshold of 30 Hz, no action was considered to be taken. Such trials were dropped from further analysis. Reaction times were calculated as time from stimulus onset to decision (either subpopulation reaches the threshold). The ‘slow’ and ‘fast’ trials were categorized as reaction times ≥75th percentile (314.5 ms) and reactions time <50 th percentile (196.0 ms), respectively, of the reaction time distributions. The firing rates of the CBGT nuclei during the reaction times were used for prediction analysis as discussed in our description of single-trial response estimation below.

### Corticostriatal weight plasticity

The corticostriatal weights are modified by a dopamine-mediated STDP rule, where the phasic dopamine is modulated by reward prediction error. The internal estimate of the reward is calculated at every trial by a Q-learning algorithm and is subtracted from the reward associated with the experimental paradigm to yield a trial-by-trial estimate of the reward prediction error. The effect of dopaminergic release is receptor dependent; a rise in dopamine promotes potentiation for dSPNs and depression for iSPNs. The degree of change in the weights is dependent on an eligibility trace, which is proportional to the coincidental presynaptic (cortical) and postsynaptic (striatal) firing rates. The STDP rule is described in detail in Vich et al., 2020 as well as in the appendix.

### In silico experimental design

We follow the paradigm of a two-arm bandit task, where the CBGT network learns to consistently choose the rewarded action until the block changes (i.e., the reward contingencies switch), at which point the CBGT network relearns the rewarded action (reversal learning). Each session consists of 40 trials with a block change every 10 trials. The reward probabilities represent a conflict of (75%, 25%); that is, in a left block, 75% of the left actions are rewarded, whereas 25% of the right actions are rewarded. The inter-trial-interval in network time is fixed to 600 ms.

To maximize the similarity between the CBGT network simulations and our human data, we randomly varied the initialization of the network such that neurons with a specific connection probability were randomly chosen for each simulated subject, with the background input to the nuclei for each simulated subject as a mean-reverting random walk (noise was drawn from the normal distribution N(0,1)). These means are listed in Supplementary file 6.

### Participants

Four neurologically healthy adult humans (two female, all right-handed, 29–34 years old) were recruited and paid $30 per session, in addition to a performance bonus and a bonus for completing all nine sessions. These participants were recruited from the local university population.

All procedures were approved by the Carnegie Mellon University Institutional Review Board (Approval Code: 2018⁢_⁢00000195). All research participants provided informed consent to participate in the study and consent to publish any research findings based on their provided data.

### Experimental design

The experiment used male and female Greebles (Gauthier and Tarr, 1997) as selection targets. Participants were first trained to discriminate between male and female Greebles to prevent errors in perceptual discrimination from interfering with selection on the basis of value. Using a two-alternative forced choice task, participants were presented with a male and female Greeble and asked to select the female, with the male and female Greeble identities resampled on each trial. Participants received binary feedback regarding their selection (correct or incorrect). This criterion task ended after participants reached 95% accuracy. After reaching perceptual discrimination criterion for each session, each participant was tested under nine reinforcement learning conditions composed of 300 trials each, generating 2700 trials per participant in total. Data were collected from four participants in accordance with a replication-based design, with each participant serving as a replication experiment. Participants completed these sessions in randomized order. Each learning trial presented a male and female Greeble (Gauthier and Tarr, 1997), with the goal of selecting the gender identity of the Greeble that was most rewarding. Because individual Greeble identities were resampled on each trial, the task of the participant was to choose the gender identity rather than the individual identity of the Greeble, which was most rewarding.

Probabilistic reward feedback was given in the form of points drawn from the normal distribution N(μ=3,σ=1) and converted to an integer. These points were displayed at the center of the screen. For each run, participants began with 60 points and lost one point for each incorrect decision. To promote incentive compatibility (Rosenzweig and Evenson, 1977), participants earned a cent for every point earned. Reaction time was constrained such that participants were required to respond within between 0.1 s and 0.75 s from stimulus presentation. If participants responded in ≤0.1 s, ≥0.75 s, or failed to respond altogether, the point total turned red and decreased by 5 points. Each trial lasted 1.5 s and reward feedback for a given trial was displayed from the time of the participant’s response to the end of the trial. To manipulate change point probability, the gender identity of the most rewarding Greeble was switched probabilistically, with a change occurring every 10, 20, or 30 trials, on average. To manipulate the belief in the value of the optimal target, the probability of reward for the optimal target was manipulated, with p set to 0.65, 0.75, or 0.85. Each session combined one value of p with one level of volatility, such that all combinations of change point frequency and reward probability were imposed across the nine sessions. Finally, the position of the high-value target was pseudo-randomized on each trial to prevent prepotent response selections on the basis of location.

### Behavioral analysis

Statistical analyses and data visualization were conducted using custom scripts written in R (R Foundation for Statistical Computing, version 3.4.3) and Python (Python Software Foundation, version 3.5.5). Scripts are publicly available (Bond et al., 2023).

Binary accuracy data were submitted to a mixed effects logistic regression analysis with either the degree of conflict (the probability of reward for the optimal target) or the degree of volatility (mean change point frequency) as predictors. The resulting log-likelihood estimates were transformed to likelihood for interpretability. RT data were log-transformed and submitted to a mixed effects linear regression analysis with the same predictors as in the previous analysis. To determine if participants used ideal observer estimates to update their behavior, two more mixed effects regression analyses were performed. Estimates of change point probability and the belief in the value of the optimal target served as predictors of reaction time and accuracy across groups. As before, we used a mixed logistic regression for accuracy data and a mixed linear regression for reaction time data.

### Estimating evidence accumulation using drift diffusion modeling

To assess whether and how much the ideal observer estimates of change point probability (Ω) and the belief in the value of the optimal target (Δ⁢B) (Nassar et al., 2010; Bond et al., 2021) updated the rate of evidence accumulation (v), we regressed the change point-evoked ideal observer estimates onto the decision parameters using hierarchical drift diffusion model (HDDM) regression (Wiecki et al., 2013). These ideal observer estimates of environmental uncertainty served as a more direct and continuous measure of the uncertainty we sought to induce with our experimental manipulations. Using this more direct approach, we pooled change point probability and belief across all conditions and used these values as our predictors of drift rate and boundary height. Responses were accuracy-coded, and the belief in the difference between targets values was transformed to the belief in the value of the optimal target ($\Delta \,B_{\text{optimal(t)}}=B_{\text{optimal(t)}}-B_{\text{suboptimal(t)}}$). This approach allowed us to estimate trial-by-trial covariation between the ideal observer estimates and the decision parameters.

To find the models that best fit the observed data, we conducted a model selection process using deviance information criterion (DIC) scores. A lower DIC score indicates a model that loses less information. Here, a difference of ≤ 2 points from the lowest-scoring model cannot rule out the higher scoring model; a difference of 3–7 points suggests that the higher scoring model has considerably less support; and a difference of 10 points suggests essentially no support for the higher scoring model (Spiegelhalter et al., 2002; Burnham and Anderson, 1998). We evaluated the DIC scores for the set of fitted models relative to an intercept-only regression model (DIC*_intercept_* - DIC*_modeli_*).

### MRI data acquisition

Neurologically healthy human participants (N = 4, two females) were recruited. Each participant was tested in nine separate imaging sessions using a 3T Siemens Prisma scanner. Session 1 included a set of anatomical and functional localizer sequences (e.g., visual presentation of Greeble stimuli with no manual responses, and left vs. right button responses to identify motor networks). Sessions 2–10 collected five functional runs of the dynamic two-armed bandit task (60 trials per run). Male and female ‘Greebles’ served as the visual stimuli for the selection targets (Gauthier and Tarr, 1997), with each presented on one side of a central fixation cross. Participants were trained to respond within 1.5 s.

To minimize the convolution of the hemodynamic response from trial to trial, inter-trial intervals were sampled according to a truncated exponential distribution with a minimum of 4 s between trials, a maximum of 16 s, and a rate parameter of 2.8 s. To ensure that head position was stabilized and stable over sessions, a CaseForge head case was customized and printed for each participant. The task-evoked hemodynamic response was measured using a high spatial (2 mm^3^ voxels) and high temporal (750 ms TR) resolution echo planar imaging approach. This design maximized recovery of single-trial evoked BOLD responses in subcortical areas, as well as cortical areas with higher signal-to-noise ratios. During each functional run, eye-tracking (EyeLink, SR Research Inc), physiological signals (ECG, respiration, and pulse oximetry via the Siemens PMU system) were also collected for tracking attention and for artifact removal.

### Preprocessing

fMRI data were preprocessed using the default fMRIPrep pipeline (Esteban et al., 2019), a standard toolbox for fMRI data preprocessing that is robust to variations in scan acquisition protocols and minimal user manipulation.

### Single-trial response estimation

We used a univariate GLM to estimate within-participant trial-wise responses at the voxel level. Specifically, for each fMRI run, preprocessed BOLD time series were regressed onto a design matrix, where each task trial corresponded to a different column, and was modeled using a boxcar function convolved with the default hemodynamic response function given in SPM12. Thus, each column in the design matrix estimated the average BOLD activity within each trial. In order to account for head motion, the six realignment parameters (three rotations, three translations) were included as covariates. In addition, a high-pass filter (128 s) was applied to remove low-frequency artifacts. Parameter and error variance were estimated using the RobustWLS toolbox, which adjusts for further artifacts in the data by inversely weighting each observation according to its spatial noise (Diedrichsen and Shadmehr, 2005).

Finally, estimated trial-wise responses were concatenated across runs and sessions and then stacked across voxels to give a matrix, ${\hat{\beta }}_{t,v}$, of *T* (trial estimations) × *V* (voxels) for each participant.

### Single-trial response prediction

A machine learning approach was applied to predict left/right Greeble choices from the trial-wise responses. First, using the trial-wise hemodynamic responses, we estimated the contrast in neural activation when the participant made a left versus right selection. A LASSO-PCR classifier (i.e., an L1-constrained principal component logistic regression) was estimated for each participant according to the below procedure. We should note that the choice of LASSO-PCR was based on prior work building reliable classifiers from whole-brain-evoked responses that maximizes inferential utility (see Meissner et al., 2011). This approach is used in case of over-parameterization, as when there are more voxels than observations, and relies on a combination of dimensionality reduction and sparsity constraints to find the effective complexity of a model.

First, a singular value decomposition (SVD) was applied to the input matrix X:

(1) $$X=USV^{T}\;,$$

where the product matrix Z=U⁢S represents the principal component scores, that is, the values of X projected into the principal component space, and $V^{T}$ an orthogonal matrix whose rows are the principal directions in feature space. Then the binary response variable y (left/right choice) was regressed onto Z, where the estimation of the β coefficients is subject to an L1 penalty term C in the objective function:

(2) $$\hat{\beta }=arg\min_{\beta }\frac{1}{2}\beta^{T}\beta +C\sum_{i=1}^{N}\log(\exp(-y_{i}(Z_{i}^{T}\beta ))+1)\;,$$

where β and *Z* include the intercept term, $y_{i}=\left\{-1,1\right\}$, and *N* is the number of observations.

Projection of the estimated $\hat{\beta }$ coefficients back to the original feature (voxel) space was done to yield a weight map $\hat{w}=V\,\hat{\beta }$, which in turn was used to generate final predictions $\hat{y}$:

(3) $$\hat{y}=\frac{1-e^{-x\cdot \hat{w}}}{1+e^{-x\cdot \hat{w}}}\;,$$

where x denotes the vector of voxel-wise responses for a given trial (i.e., a given row in the X matrix). When visualizing the resulting weight maps, these were further transformed to encoded brain patterns. This step was performed to aid in correct interpretation in terms of the studied brain process because doing this directly from the observed weights in multivariate classification (and regression) models can be problematic (Winkler et al., 2015).

Here, the competition between left–right neural responses decreases classifier decoding accuracy as neural activation associated with these actions becomes less separable. Therefore, classifier prediction serves as a proxy for response competition. To quantify uncertainty from this, we calculated the Euclidean distance of these decoded responses $\hat{y}$ from the statistically optimal choice on a given trial, o⁢p⁢t⁢_⁢c⁢h⁢o⁢i⁢c⁢e. This yielded a trial-wise uncertainty metric derived from the decoded competition between neural responses.

(4) $$\hat{U}=d(\hat{y},opt\_choice).$$

The same analytical pipeline was used to calculate single-trial responses for simulated data with a difference that trial-wise average firing rates of all nuclei from the simulations were used instead of fMRI hemodynamic responses.

### Robustness analysis

To test whether our key effects were robust to variation in parameter schemes, 300 networks were sampled using Latin Hypercube Sampling (LHS), as also described in Vich et al., 2022. From this, we chose two network configurations with biologically plausible parameters, one with slower and faster reaction times (‘fast’ and ‘slow’ networks; upper and lower quartile of the reaction time distributions). We then repeated our key analyses for these two network configurations as shown in Figure 2—figure supplement 1. We show that these adaptive network effects are robust over a range of parameter configurations, so long as the network generates firing rates are biologically plausible.

### Neuron model

We used an integrate-and-fire-or-burst model that models the membrane potential V⁢(t) as

(5) $$C\frac{dV}{dt}=-g_{L}(V(t)-V_{L})-g_{T}h(t)H(V(t)-V_{h})(V(t)-V_{T})-I_{syn}(t)-I_{ext}(t)$$

$$\frac{dh}{dt}=\left\{ \begin{array}{ll} -h(t)/\tau_{h}^{-} & \text{, when }V(t)\geq V_{h}\\ (1-h(t))/\tau_{h}^{+} & \text{, when }V(t)<V_{h} \end{array}\right.$$

where $g_{L}$ represents the leak conductance, $V_{L}$ is the leak reversal potential and the first term $g_{L}\,\left(V\,\left(t\right)-V_{L}\right)$ is the leak current; the next term is a low threshold $C\,a^{2+}$ current with maximum conductance $g_{T}$, gating variable h⁢(t), and reversal potential $V_{T}$, which activates when $V(t)>V_{h}$ due to the Heaviside function $H;\;I_{syn}$ is the synaptic current and $I_{e\,x\,t}$ is the external current. This neuron model is capable of producing post-inhibitory bursts, regulated by the gating variable that decays with the time constant $\tau_{h}^{-}$, when the membrane potential reaches a certain threshold $V_{h}$ and rises with time constant $\tau_{h}^{+}$. However, when $g_{T}$ is set to zero, the model reduces to a leaky integrate and fire neuron. Currently, we model GPe and STN neuronal populations with bursty neurons and the remaining neuronal populations with leaky integrate-and-fire neurons, all with conductance-based synapses.

The synaptic current $I_{s\,y\,n}\,\left(t\right)$ consists of three components, two excitatory currents corresponding to AMPA and NMDA receptors and one inhibitory current corresponding to GABA receptors, and is calculated as below:

$$I_{syn}=g_{\text{AMPA}}s_{\text{AMPA}}(t)(V(t)-V_{E})+\frac{g_{\text{NMDA}}s_{\text{NMDA}}(t)(V(t)-V_{E})}{1+e^{-0.062V(t)/3.57}}+g_{\text{GABA}}s_{\text{GABA}}(t)(V(t)-V_{I})$$

where $g_{\text{i}}$ represents the maximum conductance corresponding to the receptor i∈ {AMPA, NMDA, GABA}, $V_{I}$ and $V_{E}$ represent the excitatory and inhibitory reversal potentials, and $s_{\text{i}}$ represents the gating variable for each current, with dynamics given by

$$\frac{ds_{\text{AMPA}}}{dt}=\sum_{j}\delta (t-t_{j})-\frac{s_{\text{AMPA}}}{\tau_{\text{AMPA}}}$$

$$\frac{ds_{\text{NMDA}}}{dt}=\alpha (1-s_{\text{NMDA}})\sum_{j}\delta (t-t_{j})-\frac{s_{\text{NMDA}}}{\tau_{\text{NMDA}}}$$

$$\frac{ds_{\text{GABA}}}{dt}=\sum_{j}\delta (t-t_{j})-\frac{s_{\text{GABA}}}{\tau_{\text{GABA}}}$$

The gating variables for AMPA and GABA act as leaky integrators that are increased by all incoming spikes, with an additional constraint for NMDA that ensures that the maximum value of $s_{\text{NMDA}}$ remains below 1.

The values of neuronal parameters for all of the nuclei are listed in Supplementary file 3, external inputs to the CBGT nuclei are listed in Supplementary file 4, synaptic parameter values are listed in Supplementary file 5, connectivity types and probabilities are listed in Supplementary file 6, and the number of neurons in each CBGT population is shown in Supplementary file 7.

### Spike timing-dependent plasticity rule

The plasticity rule we use is a dopamine-modulated STDP rule also described in Vich et al., 2020. All the values of the relevant parameters are listed in Supplementary file 8. The weight update of a corticostriatal synapse is controlled by three factors: (1) an eligibility trace, (2) the type of the striatal neuron (iSPN/dSPN), and (3) the level of dopamine.

To compute the eligibility (E) for a given synapse, an activity trace of each neuron in the presynaptic and postsynaptic populations is tracked via the equations

$$\tau_{PRE}\frac{dA_{PRE}}{dt}=\Delta_{PRE}X_{PRE}(t)\;-\;A_{PRE}(t)$$

$$\tau_{POST}\frac{dA_{POST}}{dt}=\Delta_{POST}X_{POST}(t)\;-\;A_{POST}(t)$$

where $X_{P\,R\,E},X_{P\,O\,S\,T}$ are spike trains, such that $A_{P\,R\,E}$ and $A_{P\,O\,S\,T}$ maintain a filtered record of synaptic spiking of the pre/post neuron, respectively, with spike impact parameters $\Delta_{P\,R\,E},\Delta_{P\,O\,S\,T}$ and time constants $\tau_{P\,R\,E},\tau_{P\,O\,S\,T}$.

If the postsynaptic spike follows the spiking activity of the presynaptic population closely enough in time, then the eligibility variable E increases and allows for plasticity to occur. On the other hand, if a presynaptic spike follows the spiking activity of the postsynaptic population, then E decreases. In the absence of any activity and spikes, the eligibility trace decays to zero with a time constant $\tau_{E}$. Putting these effects together, we obtain the equation

$$\tau_{E}\,\frac{d\,E}{d\,t}=X_{P\,O\,S\,T}\,\left(t\right)\,A_{P\,R\,E}\,\left(t\right)-X_{P\,R\,E}\,\left(t\right)\,A_{P\,O\,S\,T}\,\left(t\right)-E.$$

The synaptic weight update depends on the dopamine receptor type of the striatal neuron; that is, if the neuron is a dSPN or iSPN. We assume that a phasic dopamine release promotes long-term potentiation (LTP) in dSPNs and long-term depression (LTD) in iSPNs. This factor is indicated by the learning rate parameter $\alpha_{w}$, which is set to a positive value for dSPNs and a negative value for iSPNs. The weight update dynamics is given by

(6) $$\frac{dw}{dt}=[\alpha_{w-X}E(t)f_{X}(K_{DA})(W_{max}^{X}-w){]}^{+}+[\alpha_{w-X}E(t)f_{X}(K_{DA})(w-W_{min}){]}^{-}$$

where X∈ {dSPN, iSPN} with $\alpha_{w-dSPN}\;>\;0$ and $\alpha_{w-iSPN}\;<\;0$. Here, the weights of the corticostriatal synapses are bounded between the maximal value $W_{m\,a\,x}^{X}$, which depends on the SPN type, and a minimal value of $W_{m\,i\,n}=0.001$. The precise values used for all relevant parameters are listed in Supplementary file 3.

In the weight update rule (6), $K_{D\,A}$ represents the dopamine level present. This quantity changes as a result of phasic release of dopamine (increments of size $D\,A_{i\,n\,c}$), which is correlated to the reward prediction error encountered in the environment. We define a parameter $C_{s\,c\,a\,l\,e}$ that sets the scaling between the reward prediction error and the amount of dopamine released, and $K_{D\,A}$ obeys the equation

$$\tau_{D\,O\,P}\,\frac{K_{D\,A}}{d\,t}=C_{s\,c\,a\,l\,e}\,\left(D\,A_{i\,n\,c}\,\left(t\right)-K_{D\,A}\right)\,\delta \,\left(t\right)-K_{D\,A},$$

where

$$D\,A_{i\,n\,c}\,\left(t\right)=r\,\left(t\right)-Q_{c\,h\,o\,s\,e\,n}\,\left(t\right)$$

for reward r⁢(t) and expected value $Q_{c\,h\,o\,s\,e\,n}\,\left(t\right)$ of the chosen action. Trial-by-trial estimates of the values of the actions (left/right) are maintained by a simple Q-update rule:

$$Q_{a}\,\left(t+1\right)=Q_{a}\,\left(t\right)+\alpha_{q}\,\left(r\,\left(t\right)-Q_{a}\,\left(t\right)\right)$$

where a∈ {left, right} and where $\alpha_{q}$ represents the learning rate of the Q-values.

Finally, the function $f_{X}\,\left(K_{D\,A}\right)$ converts the level of dopamine into an impact on plasticity in a way that depends on the identity X of the postsynaptic neuron, as follows:

$$f_{X}\,\left(K_{D\,A}\right)=\left\{ \begin{array}{cc} K_{D\,A}, & X=d\,S\,P\,N,\\ \frac{K_{D\,A}}{c+\left|K_{D\,A}\right|}, & X=i\,S\,P\,N, \end{array}\right.$$

where c sets the dopamine level at which $f_{i\,S\,P\,N}$ reaches half-maximum. Supplementary file 8 lists the specific parameters used for the STDP rule.

## Data Availability

Behavioral data and computational derivatives are publically available here: https://github.com/kalexandriabond/competing-representations-shape-evidence-accumulation (copy archived at Bond, 2023). Raw and preprocessed hemodynamic data, in addition to physiological measurements collected for quality control, are available here: https://openneuro.org/datasets/ds004283/versions/1.0.3. The following dataset was generated: BondK RaseroJ MadanR BahugunaJ RubinJ VerstynenT 2022neurolokiOpenNeuro10.18112/openneuro.ds004283.v1.0.3PMC1062442137818943

## Additional files

Supplementary file 1. Model fits. Group-level deviance information criterion (DIC) values for regression models tested.

Supplementary file 2. Individual model fits. Participant-level deviance information criterion (DIC) values for regression models tested.

Supplementary file 3. Neuronal parameters. Neuronal parameters for each nucleus are listed in the left column, with values shown on the right. The membrane time constant: $\tau_{m}$, resting membrane potential: $V_{\text{rest}}$, threshold potential: $V_{\text{threshold}}$, leak reversal: $V_{\text{L}}$, low threshold Ca^2+^ maximal conductance: $g_{T}$, threshold potential for burst activation: $V_{\text{h}}$, reversal potential: $V_{T}$(Ca^2+^), burst duration: $\tau_{h}^{-}$, hyperpolarization duration: $\tau_{h}^{+}$.

Supplementary file 4. External inputs to CBGT nuclei. Each nucleus is listed on the left, with mean external input frequency, efficacy, and number of connections listed by receptor.

Supplementary file 5. Synaptic parameters. Parameters for the simulated synapses.

Supplementary file 6. CBGT connectivity. Connection type and probability by nucleus and receptor.

Supplementary file 7. Number of neurons in each CBGT population.

Supplementary file 8. STDP parameters.

MDAR checklist
