# Supplementary material for: Competing neural representations of choice shape evidence accumulation in humans
Source: eLife. 2023 Oct 11;12:e85223. doi: 10.7554/eLife.85223 (PMC10624421; doi:10.7554/eLife.85223)
Supplement: Supplementary file 1. — Group-level deviance information criterion (DIC) values for regression models tested. [file elife-85223-supp1.pdf]

Simulated

	ΔB	Ω	ΔDIC_{null}	DIC$_{best}$
I	v	a	-29.85 ± 12.76	-4.49 ± 5.91
II	a	v	-23.94 ± 22.56	-10.40 ± 11.22
III	$-$	v	-6.16 ± 4.24	-28.19 ± 13.62
IV	v	$-$	-22.60 ± 7.28	-11.74 ± 14.80
V	$-$	a	-7.04 ± 11.06	-27.30 ± 8.16
VI	a	$-$	-17.72 ± 21.49	-16.62 ± 11.88
VII	$-$	$-$	0.00 ± 0.00	-34.34 ± 15.97

Human

	ΔB	Ω	ΔDIC_{null}	ΔDIC_{best}
I	v	a	-14.90 ± 20.58	-1.52 ± 1.04
II	a	v	-0.44 ± 1.11	-15.99 ± 18.56
III	$-$	v	-1.47 ± 1.30	-14.96 ± 18.56
IV	v	$-$	-13.80 ± 16.61	-2.63 ± 3.62
V	$-$	a	-1.03 ± 4.46	-15.40 ± 15.60
VI	a	$-$	$1.00 \pm .071$	-17.42 ± 19.52
VII	$-$	$-$	0.00 ± 0.00	-16.43 ± 19.53

Supplementary File 1. Model fits. Group-level Deviance Information Criterion (DIC) values for regression models tested.
