# Supplementary material for: Competing neural representations of choice shape evidence accumulation in humans
Source: eLife. 2023 Oct 11;12:e85223. doi: 10.7554/eLife.85223 (PMC10624421; doi:10.7554/eLife.85223)
Supplement: Supplementary file 2. — Participant-level deviance information criterion (DIC) values for regression models tested. [file elife-85223-supp2.pdf]

	Part.	ΔB	Ω	ΔDIC_{null}	ΔDIC_{best}
I	1	v	a	0.61	-2.32
II	1	a	v	0.08	-1.79
III	1	$-$	v	-1.71	0.00
IV	1	v	$-$	1.13	-2.84
V	1	$-$	a	-0.36	-1.35
VI	1	a	$-$	1.93	-3.64
VII	1	$-$	$-$	0.00	-1.71
I	2	v	a	-9.91	-1.73
II	2	a	v	-0.69	-10.95
III	2	$-$	v	-1.17	-10.47
IV	2	v	$-$	-11.64	0.00
V	2	$-$	a	1.89	-13.52
VI	2	a	$-$	0.46	-12.10
VII	2	$-$	$-$	0.00	-11.64
I	3	v	a	-45.08	0.00
II	3	a	v	-1.85	-43.23
III	3	$-$	v	-3.07	-42.01
IV	3	v	$-$	-37.41	-7.68
V	3	$-$	a	-7.53	-37.55
VI	3	a	$-$	1.16	-46.25
VII	3	$-$	$-$	0.00	-45.08
I	4	v	a	-5.23	-2.05
II	4	a	v	0.71	-7.99
III	4	$-$	v	-0.07	-7.35
IV	4	v	$-$	-7.28	0.00
V	4	$-$	a	1.90	-9.18
VI	4	a	$-$	0.43	-7.70
VII	4	$-$	$-$	0.00	-7.28

Supplementary File 2. Individual Model fits. Participant-level Deviance Information Criterion (DIC) values for regression models tested.
