# Supplementary material for: Competing neural representations of choice shape evidence accumulation in humans
Source: eLife. 2023 Oct 11;12:e85223. doi: 10.7554/eLife.85223 (PMC10624421; doi:10.7554/eLife.85223)
Supplement: Supplementary file 3. — Neuronal parameters for each nucleus are listed in the left column, with values shown on the right. The membrane time constant: τm, resting membrane potential: Vrest, threshold potential: Vthreshold, leak reversal: VL, low threshold Ca2+ maximal conductance: gT, threshold potential for burst activation: Vh, reversal potential: VT(Ca2+), burst duration: τh-, hyperpolarization duration: τh+. [file elife-85223-supp3.pdf]

Param.	Unit	Cx	CxI	dSPN	iSPN	FSI	GPe	STN	Thalamus
τ_m	ms	20	10	20	20	10	20	20	27.78
V_{rest}	mV	-70	-70	-70	-70	-70	-70	-70	-70
$V_{\text{threshold}}$	mV	-50	-50	-50	-50	-50	-50	-50	-50
V_{L}	mV	-55	-55	-55	-55	-55	-55	-55	-55
g_T	mS/cm^2	0	0	0	0	0	0.06	0.06	0
V_{h}	mV	-60	-60	-60	-60	-60	-60	-60	-60
V_T	mV	120	120	120	120	120	120	120	120
τ_h^-	ms	20	20	20	20	20	20	20	20
τ_h^+	ms	100	100	100	100	100	100	100	100

Supplementary File 3. Neuronal parameters. Neuronal parameters for each nucleus are listed in the left column, with values shown on the right. The membrane time constant: τ_m, resting membrane potential: V_{rest}, threshold potential: $V_{\text{threshold}}$, leak reversal: V_{L}, low threshold Ca^{2+} maximal conductance: g_T, threshold potential for burst activation: V_{h}, reversal potential: V_T(Ca^{2+}, burst duration: τ_h^-, hyperpolarization duration: τ_h^+.
