# Supplementary material for: Competing neural representations of choice shape evidence accumulation in humans
Source: eLife. 2023 Oct 11;12:e85223. doi: 10.7554/eLife.85223 (PMC10624421; doi:10.7554/eLife.85223)
Supplement: Supplementary file 4. — Each nucleus is listed on the left, with mean external input frequency, efficacy, and number of connections listed by receptor. [file elife-85223-supp4.pdf]

Population	Receptor	External input freq.	External input efficacy	No. external connections
CxI	AMPA	3.7	1.2	640
Cx	AMPA	2.3	2.0	800
dSPN	AMPA	1.3	4.0	800
iSPN	AMPA	1.3	4.0	800
FSI	AMPA	3.6	1.55	800
GPi	AMPA	0.8	5.9	800
GPe	AMPA	4	2.0	800
GPe	GABA	2	2.0	2000
STN	AMPA	4.45	1.65	800
Thalamus	AMPA	2.2	2.5	800

Supplementary File 4. External inputs to CBGT nuclei. Each nucleus is listed on the left, with mean external input frequency, efficacy, and number of connections listed by receptor.
