# Supplementary material for: Competing neural representations of choice shape evidence accumulation in humans
Source: eLife. 2023 Oct 11;12:e85223. doi: 10.7554/eLife.85223 (PMC10624421; doi:10.7554/eLife.85223)
Supplement: Supplementary file 5. — Parameters for the simulated synapses. [file elife-85223-supp5.pdf]

Parameter	Unit	Value
τ_{AMPA}	ms	2
V_{E}	mV	0
τ_{NMDA}	ms	100
τ_{GABA}	ms	5
V_{I}	mV	-70
α	-	0.6332

Supplementary File 5. Synaptic parameters. Parameters for the simulated synapses.
