# Supplementary material for: Competing neural representations of choice shape evidence accumulation in humans
Source: eLife. 2023 Oct 11;12:e85223. doi: 10.7554/eLife.85223 (PMC10624421; doi:10.7554/eLife.85223)
Supplement: Supplementary file 6. — Connection type and probability by nucleus and receptor. [file elife-85223-supp6.pdf]

Connection Type	Connection Prob.	g (nS)	Receptor
Cx-dSPN	1.0	0.015	AMPA
Cx-dSPN	1.0	0.02	NMDA
Cx-iSPN	1.0	0.015	AMPA
Cx-iSPN	1.0	0.02	NMDA
Cx-FSI	1.0	0.43	AMPA
Cx-Th	1.0	0.025	AMPA
Cx-Th	1.0	0.035	NMDA
Cx-Cx	0.13	0.0127	AMPA
Cx-Cx	0.13	0.08	NMDA
Cx-CxI	0.0725	0.113	AMPA
Cx-CxI	0.0725	0.525	NMDA
CxI-Cx	0.5	1.05	GABA
CxI-CxI	1.0	1.075	GABA
dSPN-dSPN	0.45	0.28	GABA
dSPN-iSPN	0.45	0.28	GABA
dSPN-GPi	1.0	2.09	GABA
iSPN-iSPN	0.45	0.28	GABA
iSPN-dSPN	0.5	0.28	GABA
iSPN-GPe	1.0	4.07	GABA
FSI-FSI	1.0	3.2583	GABA
FSI-dSPN	1.0	1.77	GABA
FSI-iSPN	1.0	1.66	GABA
GPe-GPe	0.067	1.75	GABA
GPe-STN	0.067	0.35	GABA
GPe-GPi	1.0	0.058	GABA
STN-GPe	0.1617	0.07	AMPA
STN-GPe	0.1617	1.51	NMDA
STN-GPi	1.0	0.038	GABA
GPi-Th	1.0	0.033	GABA
Th-dSPN	1.0	0.38	AMPA
Th-iSPN	1.0	0.38	AMPA
Th-FSI	0.83	0.1	AMPA
Th-Cx	0.83	0.03	NMDA

Supplementary File 6. CBGT connectivity. Connection type and probability by nucleus and receptor.
