# Supplementary material for: Competing neural representations of choice shape evidence accumulation in humans
Source: eLife. 2023 Oct 11;12:e85223. doi: 10.7554/eLife.85223 (PMC10624421; doi:10.7554/eLife.85223)
Supplement: Supplementary file 7. [file elife-85223-supp7.pdf]

Population	No. neurons
Cx	204
CxI	186
dSPN	75
iSPN	75
FSI	75
GPe	750
GPi	75
STN	750
Th	75

Supplementary File 7. Number of neurons in each CBGT population.
