# Supplementary material for: Competing neural representations of choice shape evidence accumulation in humans
Source: eLife. 2023 Oct 11;12:e85223. doi: 10.7554/eLife.85223 (PMC10624421; doi:10.7554/eLife.85223)
Supplement: Supplementary file 8. [file elife-85223-supp8.pdf]

Parameter	Value
Δ_{PRE}	0.8
Δ_{POST}	0.04
τ_{PRE}	15
τ_{POST}	6
τ_{E}	100
α_{w-dSPN}	39.5
α_{w-iSPN}	-38.2
W_{max}^{dSPN}	0.055
W_{max}^{iSPN}	0.035
W_{min}	0.001
c	2.5
τ_{DOP}	2.0
α_{q}	0.6
C_{scale}	85

Supplementary File 8. STDP parameters.
