# Supplementary material for: Competing neural representations of choice shape evidence accumulation in humans
Source: eLife. 2023 Oct 11;12:e85223. doi: 10.7554/eLife.85223 (PMC10624421; doi:10.7554/eLife.85223)
Supplement: Peer review history

# Editor report: Editor's evaluation

**DOI:** 10.7554/eLife.85223.sa0

**Authors:** Tobias H Donner

**Affiliations:** https://ror.org/01zgy1s35 University Medical Center Hamburg-Eppendorf Germany

This valuable study presents solid evidence for how change in reward contingency in the environment affects the dynamics of a realistic large-scale neural circuit model, human choice behavior, and fMRI responses. This study could be of interest to scientists studying the neural and computational bases of adaptive behavior.

# Decision letter

**DOI:** 10.7554/eLife.85223.sa1

**Affiliations:** https://ror.org/01zgy1s35 University Medical Center Hamburg-Eppendorf Germany

Our editorial process produces two outputs: (i) public reviews designed to be posted alongside the preprint for the benefit of readers; (ii) feedback on the manuscript for the authors, including requests for revisions, shown below. We also include an acceptance summary that explains what the editors found interesting or important about the work.

**Decision letter after peer review:**

[Editors’ note: the authors submitted for reconsideration following the decision after peer review. What follows is the decision letter after the first round of review.]

Thank you for submitting your article "Competing neural representations of choice shape evidence accumulation in humans" for consideration by *eLife*. Your article has been reviewed by 2 peer reviewers, and the evaluation has been overseen by a Reviewing Editor and Floris de Lange as the Senior Editor. The reviewers have opted to remain anonymous.

The reviewers have discussed their reviews with one another, and the Reviewing Editor has drafted this to help you prepare a revised submission.

Essential revisions:

1) Overall, reviewers felt that the study would benefit from (i) the derivation of more (and/or more specific) model predictions from the neural circuit model (ii) more in-depth analyses of the fMRI data, and (iii) further steps to link DDM, circuit model, and fMRI data.

For e.g. model-based analyses could be performed to elucidate the activity dynamics and link that to the circuit model. This could perhaps be done e.g. with slow and fast responses, and identify brain regions with ramping activities over time as in the model (e.g. Supp. Figure 1).

2) Please clarify the correlation between drift and model uncertainty (figure 2E) and drift and fMRI classifier uncertainty (figure 4B), and explain the largely weaker association between classier uncertainty and drift rate (Figure 4B), and the weak reaction time effects (Supp. Figure 2).

We assume that the posterior probability distributions shown are obtained from the HDDM single-trial regression mode. Should the spiking NN model and the fMRI results not be compared to a simpler null model (e.g. with a weighted average of past responses) to know how much it is an improvement over previous work?

3) Please discuss why the model's cortical neurons had no contralateral encoding, unlike the fMRI data.

4) Please clarify why the prediction of CBGT network choices (lines 130-131) is not 100%:

Presumably, all the relevant information (firing rates of all regions modelled) together should perfectly predict the choice. (How crucial is the choice of the LASSO-PCR classifier over other classifiers?)

5) Please clarify the terminology:

– Throughout, there are aspects of the manuscript that make it hard for the reader to follow. For instance, in Figure 1 the terms D1-SPN/D2-SPN are used interchangeably with dSPN and iSPN, and the legend and the text differ in what the time = 0 indicates (stimulus or decision).

– Change of mind in the literature is now more linked to a trial change in (impending) choices, a more recent research area (e.g. Resulaj et al., Nat. 2009), and the phrase change of mind is not as suitable for use in this work. I would replace such phrases with phrases like adaptive decision/choice (learned over trials) or similar.

6) Please revise the abstract: It is currently too general and vague.

*Reviewer #1 (Recommendations for the authors):*

Specific comments and recommendations:

1. Further analysis of the fMRI data may be needed. E.g. model-based analysis to elucidate the activity dynamics and link that to the biophysical data. This could perhaps be done e.g. with slow and fast responses, and identify brain regions with ramping activities over time as in the model (e.g. Supp. Figure 1).

2. Provide an explanation for the (order of magnitude) weaker association between classier uncertainty and drift rate (by participants) for human participants (Figure 4B), and the weak reaction time effects in Supp. Figure 2.

3. Discuss why the model's cortical neurons had no contralateral encoding, unlike in the neuroimaging data.

4. Change of mind in the literature is now more linked to within trial change in (impending) choices, a more recent research area (e.g. Result et al., Nat. 2009), and the phrase change of mind is not as suitable for use in this work. I would replace such phrases with phrases like adaptive decision/choice (learned over trials) or similar.

5. Supp. Figure 4. It would have been clearer to show the activity dynamics over time for the key brain regions, e.g. with fast and slow decisions. Is there actually ramping of activity over time? Could brain regions linked to dopaminergic activity be obtained and related to that in model simulations?

6. Supp. Figure 5A. Why are the weights for GPi so strong, but not much in humans?

7. Supp. Table 1. Why is the accuracy so low, hovering around the chance level?

8. Lines 470-471. Is there any non-decision latency (e.g. signal transduction and motor preparation) to bridge from decision time to reaction time?

9. Lines 604-605. The use of the classifier LASSO-PCR was not justified.

*Reviewer #2 (Recommendations for the authors):*

I will focus mostly on the behavioral task and HDDM model, as well as the link between behavior and the in silico model. My expertise does not lie in evaluating the details of the spiking neural network model, or the processing of fMRI data.

Specific questions:

– One methodological concern/clarification: the correlation between drift and model uncertainty (figure 2E) and drift and fMRI classifier uncertainty (figure 4B) seems the crucial test of the similarity between brain, behavior, and model. However, drift is not usually fit on a single trial level. So, I think the distributions shown are the posteriors from the HDDM regression model – but if so, should the spiking NN model + the fMRI results not be compared to a simpler null model (e.g. with a weighted average of past responses) to know how much it's an improvement over previous work?

– A block switch every 10 trials seems very easy to learn (i.e. simply by counting). Did any of the four humans take this strategy?

– I'm a bit puzzled the prediction of CBGT network choices (lines 130-131) is not 100%, since presumably all the relevant information (firing rates of all regions modelled) together should perfectly predict the choice. How crucial is the choice of the LASSO-PCR classifier (over other classifiers)?

[Editors’ note: further revisions were suggested prior to acceptance, as described below.]

Thank you for resubmitting the paper entitled "Competing neural representations of choice shape evidence accumulation in humans" for further consideration by *eLife*. Your revised article has been evaluated by a Senior Editor and a Reviewing Editor. We are sorry to say that we have decided that this submission will not be considered further for publication by *eLife*.

We appreciate your efforts in trying to address the reviewers' points. While the reviewers were satisfied with several of your responses, there are remaining issues. Most importantly, both reviewers felt that the study, while useful, appears a bit preliminary and the evidence in support of your conclusions remains incomplete.

*Reviewer #1 (Recommendations for the authors):*

The overall responses from the authors were mainly satisfactory, but further concerns remain.

1. Perhaps the authors can replace the spiking neural network model with simpler network-RL-based theoretical models that are more suitably linked and optimised to BOLD-fMRI data or remove the spiking neural network model which can be used and validated in their subsequent future work based on more precise neural recording.

2. Both CBGT and DDMs' parameters did not seem to be optimised. The authors' justification is that model optimisation based on experimental data could lead to 'circular' inference. Although I can understand this to some extent, especially with physiologically constrained model, I am not sure whether I fully agree with this claim. In any case, the authors should at least summarise clearly which CBGT and DDM parameters were free, which were constrained, and which were tuned – a summary table may help.

Overall, this work is interesting and could potentially contribute to the computational modelling and neuroscience of adaptive choice behaviour. However, a major component of the work, on the CBGT modelling, seems somewhat premature.

*Reviewer #2 (Recommendations for the authors):*

I thank the authors for clarifying various technical details.

While this is solid work, I am still unsure as to the main insights we can draw from this paper itself. The main argument for using the fine-scale model to account for fMRI data is that it sets the stage for future work, which will compare these model predictions with mouse data. As much as I understand that this is how science goes, the result for this paper is that linking fMRI and the detailed model is a bit strange (and makes it much harder to draw conclusions from). For the general readership of *eLife*, I'm not sure if the current insights are very helpful before knowing the results of the more fine-grained data that are currently being collected.

[Editors’ note: further revisions were suggested prior to acceptance, as described below.]

Thank you for choosing to send your work entitled "Competing neural representations of choice shape evidence accumulation in humans" for consideration at *eLife*.

Your letter of appeal has now been considered by myself as Senior Editor and Tobias as Reviewing Editor, and we had the time to discuss it. We apologize for the delay in responding to your appeal: it reached us when Tobias was just leaving for vacation; after his return, Floris was not available for some time.

After careful consideration, we are now prepared to receive a revised submission (with no guarantees of acceptance), which implements the revision plan you have outlined in your appeal.

We would like to stress that we do see the value of model-based neuroimaging in general, including work that uses biophysically detailed circuit models. We neither see the need for a general justification of such approaches, nor for references to other work using circuit modelling of fMRI data, in your paper. What we feel does need justification, in light of the comments by both reviewers, is the choice of your specific modeling approach for these particular (task) data. We also feel that a discussion of the limitations of your approach is warranted that takes into account the concerns raised by both reviewers.

# Reply: Author response

**DOI:** 10.7554/eLife.85223.sa2

[Editors’ note: The authors appealed the original decision. What follows is the authors’ response to the first round of review.]

Essential revisions:

1) Overall, reviewers felt that the study would benefit from (i) the derivation of more (and/or more specific) model predictions from the neural circuit model (ii) more in-depth analyses of the fMRI data, and (iii) further steps to link DDM, circuit model, and fMRI data.

For e.g. model-based analyses could be performed to elucidate the activity dynamics and link that to the circuit model. This could perhaps be done e.g. with slow and fast responses, and identify brain regions with ramping activities over time as in the model (e.g. Supp. Figure 1).

We agree that a fine-grained analysis of intra-trial dynamics would be an ideal complement to our current approach. However, due to the timing of the trials in relation to the lag and auto-correlational structure of the BOLD response, this sort of analysis would not yield productive results, as the entire trial is encompassed by a single evoked response. Our findings are primarily focused on variations in the magnitude of the evoked response, rather than the temporal dynamics within the trial. Although we acknowledge the importance of investigating these intra-trial dynamics, this requires neurophysiological recording with much higher temporal resolution, as the limitations of fMRI restrict our ability to evaluate them.

This difference in resolution, understandably, raises the question of why we compared a model with intra-trial neuronal dynamics with the hemodynamic response. The reason we use a biologically realistic neural network model is precisely so that it can be used as a theory bridge between multiple lines of experimentation, from macroscopic BOLD responses in humans to spiking responses in rodents (ongoing collaborative work with our lab). So this approach does have clear benefits in laying the groundwork for future research that can reveal the intricacies of the underlying mechanisms. This is done using an upward mapping perspective, where lower-level implementation models represent the biophysical properties of neurons and synapses, and higher-level models capture the emergent properties of these neural networks. This strategy allows us to make predictions at different levels of abstraction, from molecular and cellular to behavioral and cognitive, by leveraging information from lower-level models to inform higher-level ones.

For example, in our ongoing work, we are using the same neural network to test our predictions about D1 and D2 optogenetic stimulation in mice. The complexity of the model is essential for this purpose, as it provides a comprehensive framework that captures the intricacies of the underlying neural mechanisms.

In the current paper, we compared fMRI findings with the predicted dynamics at a common level of abstraction. However, due to the differences in resolution between these two approaches, our comparison is necessarily coarse. Nonetheless, we think that our approach serves as a valuable foundation for future work that can reveal the subtle details of the neural processes involved.

2) Please clarify the correlation between drift and model uncertainty (figure 2E) and drift and fMRI classifier uncertainty (figure 4B), and explain the largely weaker association between classier uncertainty and drift rate (Figure 4B), and the weak reaction time effects (Supp. Figure 2).

Indeed, this is a valid point. We now acknowledge the difference in magnitude of effects observed between the simulated and human data, and we have identified four reasons for this disparity.

First, the simulated data is not subject to the same sources of noise as the human data. The human data reflects a macroscopic proxy of underlying neural dynamics and has a strong autocorrelation structure in the signal, which adds substantial variability, attenuating the magnitude of any correlational effects with behavior.

Second, the model is not susceptible to other non-task related variance that humans are likely to experience, such as fatigue or attentional lapses.

Third, we used the model to predict the associations that we would see in humans to maintain the independence of the prediction. We did not fine-tune the model using human data to avoid circular inference. Though we can now use this data for future theoretical predictions.

Lastly, for the sake of simplicity, the simulations used only one experimental condition with a deterministic frequency of a shift in the statistically optimal option, while our human experiments varied the relative value of the two options and volatility, which are stochastic. This led to increased variance in human responses, providing more information to work with but decreased precision.

Although the qualitative pattern of results was the focus of our study, we have clarified the reasons for the difference in magnitude between the human and simulated data in the Discussion section of the revised manuscript.

We assume that the posterior probability distributions shown are obtained from the HDDM single-trial regression mode.

We agree that this was not clear. Figures 2E and 4B show bootstrapped distributions of the association test (Β weight) between classifier uncertainty and drift rate, not the HDDM posteriors. We now clarify this in the figure captions.

Should the spiking NN model and the fMRI results not be compared to a simpler null model (e.g. with a weighted average of past responses) to know how much it is an improvement over previous work?

We now clarify that we compared all pairwise and single-parameter variants of the HDDM model, along with a null model predicting average responses (no change in decision policy). The drift-rate model provided the best fit to our data among these comparisons.

However, we did not test an alternative to the central hypothesis linking behavior and implementation to decision policy. This is key to our central claim in the paper, so we thank the editor and reviewer for pointing this out. To this end, we now analyze boundary height as our target variable associated with classifier uncertainty. This parameter showed no association with classifier uncertainty. We present the results of this analysis in Figure 4 – Figure Supp. 1.

3) Please discuss why the model's cortical neurons had no contralateral encoding, unlike the fMRI data.

Thank you for pointing out the need to clarify our modeling decisions. Our model of the CBGT circuit assumes that distinct populations represent unique actions and it is agnostic to the specific laterality (or any other regional localization) in the brain. Our model assumptions hold as long as the populations representing the actions are unique, regardless of hemisphere. We have clarified this in the main text.

4) Please clarify why the prediction of CBGT network choices (lines 130-131) is not 100%:

Presumably, all the relevant information (firing rates of all regions modelled) together should perfectly predict the choice. (How crucial is the choice of the LASSO-PCR classifier over other classifiers?)

With respect to the behavioral accuracy of the CBGT model, it should be noted that this was a probabilistic decision-making task. For the model, the optimal choice was rewarded 75% of the time while the other choice was rewarded 25% of the time. This means that achieving a 100% accuracy in selecting the statistically optimal choice would result in a ceiling accuracy of 75%. However, the fact that the model does not reach 100% accuracy is actually a feature, not a bug, as it reflects the network's experience of decision uncertainty. This variability is leveraged to evaluate our primary hypothesis regarding uncertainty across action channels and drift rate in the decision process.

Regarding the choice of classifier, we used the LASSO-PCR approach, a common method for building whole brain classifiers (see Wager et al. 2011), because it is a conservative approach to inference on high dimensional data. While other machine learning methods could have been used to handle high data complexity, many of them are "black box" and make interpretability more challenging. We could have also used ridge regression or elastic net combined with PCR, but these are unlikely to change the overall conclusions of our findings because of the complete independence of each principal component.

Nonetheless, we have conducted a follow-up analysis on the neuroimaging data without the LASSO regularization step, to show how a more traditional decoder model (PCR) would do.

This produced largely the same accuracy as our model with LASSO penalties, as shown in item 4 for Reviewer #1 under minor concerns. Despite the fact that the choice of classifier parameters has a minor impact on the accuracy, we have chosen to keep the LASSO-PCR model to maintain a conservative approach and improved classifier accuracy. However, our data and code are publicly available for anyone interested in exploring different classification models for predicting human choices.

Further justification for our analysis choice has been added to the Methods and Results sections.

5) Please clarify the terminology:

– Throughout, there are aspects of the manuscript that make it hard for the reader to follow. For instance, in Figure 1 the terms D1-SPN/D2-SPN are used interchangeably with dSPN and iSPN, and the legend and the text differ in what the time = 0 indicates (stimulus or decision).

We now consistently refer to direct and indirect pathway SPNs as dSPN and iSPN, respectively.

– Change of mind in the literature is now more linked to a trial change in (impending) choices, a more recent research area (e.g. Resulaj et al., Nat. 2009), and the phrase change of mind is not as suitable for use in this work. I would replace such phrases with phrases like adaptive decision/choice (learned over trials) or similar.

Thanks for pointing this out! To minimize confusion, we have adopted the term "flexible decision-making" to refer to a change of mind instead.

6) Please revise the abstract: It is currently too general and vague.

We have overhauled the abstract to increase its specificity.

Reviewer #1 (Recommendations for the authors):

Specific comments and recommendations:

1. Further analysis of the fMRI data may be needed. E.g. model-based analysis to elucidate the activity dynamics and link that to the biophysical data. This could perhaps be done e.g. with slow and fast responses, and identify brain regions with ramping activities over time as in the model (e.g. Supp. Figure 1).

Intuitively we fundamentally agree with the reviewer’s comment – what exactly is it about the fMRI responses that delineates shifts in response policies to change? It is appealing to look for something like the accumulatoresque dynamics reported by the Shadlen lab and others from neurophysiological recordings. However, we are fundamentally limited due to the nature of the hemodynamic response itself. This signal is essentially a very low-pass filtered version of aggregated neural (and non-neural) responses over the course of the entire trial. We simply do not have the temporal resolution with this signal to do the fine grained temporal analysis of fast and slow trials. What we get is essentially variation in the amplitude of the aggregate response to the entire trial. This is what is captured in our single-trial response estimates at the core of our analysis.

In fact, this limitation is why we chose to represent the macroscopic network dynamics as classifier uncertainty. This allows us to cleanly link the cognitive model results to both the behavior and the neural dynamics at the trial-by-trial level using only two variables (drift rate and classifier uncertainty).

Nonetheless, we now clarify this in the manuscript:

“In other words, the distance from the optimal target should increase with increased co-activation of circuits that represent opposing actions. The decision to model aggregate trial dynamics with a classifier stems from the limitations of the hemodynamic response that we will use next to vet the model predictions in humans. The low temporal resolution of the evoked BOLD signal makes finer-grained temporal analysis for the human data impossible, as the signal is a low-pass filtered version of the aggregate response over the entire trial. So, we chose to represent the macroscopic network dynamics as classifier uncertainty, that cleanly links the cognitive model results to both behavior and neural dynamics at the trial-by-trial level using only two variables (drift rate and classifier uncertainty). This approach allows us to directly compare model and human results.”

2. Provide an explanation for the (order of magnitude) weaker association between classier uncertainty and drift rate (by participants) for human participants (Figure 4B), and the weak reaction time effects in Supp. Figure 2.

This is an excellent point. We agree that the difference in magnitude of effects from model to data is important. There are four reasons that we observe a greater magnitude of effect in the simulated results.

First, the simulated data aren’t affected by the same sources of noise as the human data. Our model focuses on the CBGT circuit in isolation, using restricted cell types, deterministic firing properties, and a simple mapping between information and firing rate. The human data, by comparison, reflects a macroscopic proxy of underlying neural dynamics (i.e., hemodynamic response) from much larger and heterogeneous cell populations, with an indirect mapping between neural activity and signal (i.e., neurovascular coupling), and a strong autocorrelation structure to the noise in the hemodynamic signal itself. This adds in substantially more variability that would, expectedly, attenuate the magnitude of any effects in the human data.

Second, and building off of our prior point, our computational model isn’t susceptible to other non-task related variance, like level of fatigue, an attentional lapse, etc. that the humans likely experience. In fact, the MRI magnet is a less than ideal testing environment for many cognitive tasks, which can add substantial variance to behavior. Our model did not have these same contextual influences.

Third, and crucially, we only used this model to *predict* the associations we would see in humans. Using human data to fine-tune the model would invert this logic, compromising the independence of the prediction by allowing information from the empirical data to leak into the prediction. So, given that we wanted to truly test our predicted results and that fine-tuning would result in circular inference, we compare the qualitative patterns of human and model results.

Fourth, and finally, the simulations only used a single experimental condition and the frequency of a shift in the statistically optimal option (volatility) was deterministic, leading to cleaner results. Our human experiments varied the relative value of the two options (conflict) and volatility for a total of nine experimental conditions per subject (Figure 3 Figure Supp. 2). Importantly, these manipulations are stochastic, so that although all participants went through conditions with the same statistical features, no two participants experienced the same specific experimental conditions at the trial-by-trial level.

Altogether, these factors increase the variance of human responses, meaning we have more information to work with, but decreased precision. Because of this, our goal was to compare the qualitative pattern of results for the model and the humans. Nonetheless, we have clarified the reasons for this discrepancy in magnitude in the Discussion section of the revised manuscript to make these issues clear:

“Careful attention to the effect size of our correlations between channel competition and drift rate shows that the effect is substantially smaller in humans than in the model. This is not surprising and due to several factors. Firstly, the simulated data is not affected by the same sources of noise as the hemodynamic signal, whose responses can be greatly influenced by factors such as heterogeneity of cell populations and properties of underlying neurovascular coupling. Additionally, our model is not susceptible to non-task related variance, such as fatigue or lapses of attention, which the humans likely experienced. We could have fine tuned the model results based on the empirical human data, but that would contaminate the independence of our predictions. Finally, our simulations only used a single experimental condition, whereas human experiments varied the relative value of options and volatility, which led to more variance in human responses. Yet, despite these differences we see qualitative similarities in both the model and human results, providing confirmation of a key aspect of our theory.”

3. Discuss why the model's cortical neurons had no contralateral encoding, unlike in the neuroimaging data.

The reviewer brings up an important clarification that is needed about our modeling decisions. Our model of the CBGT pathways uses a simplified design of isolated “action channels”, which are generally defined as separable populations representing unique actions (e.g., left or right hand responses) (Mink, 1996). So our model is agnostic as to the true laterality of representations in the brain, so long as they are relatively distinct representations. This is, in fact, what we see in our human neuroimaging data. Neural representations of left and right actions are distinct and they compete (Figure 3C; Figure 3 Figure Supp. 4).

Indeed, the lateralization of unimanual actions is complicated in reality, with more bilateral representations for left hand actions than right hand actions (e.g., Verstynen et al. 2005). So long as the populations representing the action are unique, regardless of hemisphere, our underlying model assumptions hold.

We now clarify this point in the main text:

“A critical assumption of the canonical model is that the basal ganglia are organized into multiple "channels", mapped to specific action representations, each containing a direct and indirect pathway. It is important to note that, for the sake of parsimony, we adopt a simple and canonical model of CBGT pathways, with action channels that are agnostic as to the location of representations (e.g., lateralization), simply assuming that actions have unique population-level representations.”

4. Change of mind in the literature is now more linked to within trial change in (impending) choices, a more recent research area (e.g. Result et al., Nat. 2009), and the phrase change of mind is not as suitable for use in this work. I would replace such phrases with phrases like adaptive decision/choice (learned over trials) or similar.

We are happy to minimize confusion with this other research area. We now refer to a change of mind as flexible decision-making, as in the abstract:

“Adapting to a changing world requires flexible decisions. Previously, we showed how the evidence accumulation process driving decisions shifts when outcome contingencies change. […]”

5. Supp. Figure 4. It would have been clearer to show the activity dynamics over time for the key brain regions, e.g. with fast and slow decisions. Is there actually ramping of activity over time? Could brain regions linked to dopaminergic activity be obtained and related to that in model simulations?

See our reply to comment #1 above. Given the timing of the trials, relative to the lag and auto-correlational structure of the BOLD response, this analysis is unlikely to yield anything productive because the entire trial is subsumed under a single evoked response. Our results here are based on variation in magnitude of the evoked response. While we are sympathetic to this question, and indeed this is the goal of follow up work with neurophysiological recordings in rodents, the limitations of the fMRI signal restrict our resolution for evaluating these sorts of intra-trial dynamics.

6. Supp. Figure 5A. Why are the weights for GPi so strong, but not much in humans?

The reason for this is again due to the limitations of the BOLD response. The pallidum is heavy in iron, which substantially impacts the signal-to-noise for detecting blood oxygenation changes in the hemodynamic signal. In addition, the primary synaptic inputs into the pallidum are GABAergic. It is still somewhat controversial as to whether and how the BOLD response is sensitive to GABAergic activity. This fundamentally limits what we can resolve with the hemodyamic response in this area.

7. Supp. Table 1. Why is the accuracy so low, hovering around the chance level?

This was a dynamic task in which the best option changed probabilistically. This means that participants had to switch their responses, necessarily resulting in errors as they adapt to the new environmental contingencies. The reviewer is right – a summary statistic is likely not the best measure. We have removed that table. We now show the average evoked accuracy and RT, following the change point as the comparison.

8. Lines 470-471. Is there any non-decision latency (e.g. signal transduction and motor preparation) to bridge from decision time to reaction time?

Yes, the hierarchical drift diffusion modeling (HDDM) framework incorporates a specific estimate of non-decision influences on reaction time, known as ‘non-decision time’ (tr). This is accounted for as a static parameter in our HDDM fits.

9. Lines 604-605. The use of the classifier LASSO-PCR was not justified.

Our approach was based on prior work showing the utility of the method for whole-brain decoding (e.g. Rasero et al. 2021, Wager et al. 2011). The basic problem is that the statistical model is very high dimensional, with many more voxels than trials. So the combination of dimensionality reduction and sparsity constraints helps to reign in the complexity of the model. The beauty of the LASSO-PCR approach is that it effectively handles high model dimensionality while allowing for clear interpretability (as opposed to methods like random forests or support vector machines, which are more difficult to make mechanistic inferences from).

But the reviewer raises an interesting point. We have rerun our neuroimaging and simulation analysis without LASSO regularization for comparison (i.e., using only the dimensionality of a PCR model). The prediction accuracy and ROC curves for the models are shown in Author response image 1, with the regularized results shown in Figure 3.

**Author response image 1.** 

Looking at the hold out accuracies, regularization does improve choice prediction, with the regularized model ~1.2 times as likely to correctly classify an action. However, this effect was small (z=1.917, p=0.055).Nonetheless this is an important point. We have added further justification of our analysis choice to the Methods and the Results:

Results:

“The choice of LASSO-PCR was based on prior work building reliable classifiers from whole-brain evoked responses that maximizes inferential utility (see Wager et al. 2011). The method is used when models are over-parameterized, as when there are more voxels than observations, relying on a combination of dimensionality reduction and sparsity constraints to find the true, effective complexity of a given model. While these are not considerations with our network model, they are with the human validation experiment that we describe next. Thus we used the same classifier on our model as on our human participants to directly compare theoretical predictions and empirical observations.”

Methods:

“A Lasso-PCR classifier (i.e. an L1-constrained principal component logistic regression) was estimated for each participant according to the below procedure. We should note that the choice of LASSO-PCR was based on prior work (see Wager et al. 2011). This approach is used in case of over-parameterization, as when there are more voxels than observations, and relies on a combination of dimensionality reduction and sparsity constraints to find the effective complexity of a model.”

Reviewer #2 (Recommendations for the authors):

I will focus mostly on the behavioral task and HDDM model, as well as the link between behavior and the in silico model. My expertise does not lie in evaluating the details of the spiking neural network model, or the processing of fMRI data.

Specific questions:

– One methodological concern/clarification: the correlation between drift and model uncertainty (figure 2E) and drift and fMRI classifier uncertainty (figure 4B) seems the crucial test of the similarity between brain, behavior, and model. However, drift is not usually fit on a single trial level. So, I think the distributions shown are the posteriors from the HDDM regression model.

We agree that this was not clear. Figures 2E and 4B show bootstrapped distributions of the association test (Β weight) between classifier uncertainty and drift rate, not the HDDM posteriors. We now clarify this in the figure captions for 2E and 4B:

2E: “Bootstrapped estimates of the association between classifier uncertainty and drift rate. […]“.

4B: “Bootstrapped estimates of the association […]”.

– But if so, should the spiking NN model + the fMRI results not be compared to a simpler null model (e.g. with a weighted average of past responses) to know how much it's an improvement over previous work?

This is a correct assumption indeed. In fact, we have compared all pairwise variants of the HDDM model (i.e., an array of comparison hypotheses), along with a more rigid null model predicting average responses (Supp. Files 1 and 2). The drift-rate model shows the best fit to the observed data among this full set of model comparisons. We now make this clearer in the main text (Results):

“We compared models where single parameters changed in response to a switch, pairwise models where both parameters changed, and a null model that predicts no change in decision policy (Supp. Table 2, Supp. Table 3).”

However, we will admit that we did not compare the central *linking hypothesis* (between behavior, decision policies, and implementation systems) that is key to our central claim in the paper. To test an alternative linking hypothesis, we conducted the same analysis using boundary height as our target variable associated with classifier uncertainty in humans. We have previously shown that this parameter also adapts in response to a change in outcome uncertainty (Bond et al. 2021). Overall, this parameter fails to show an association with classifier uncertainty overall and in all but one subject, who has a weak positive association. Comparing this effect to the stronger effect association between classifier uncertainty and drift rate presents a more severe test of our primary hypothesis.

We now show the evoked response and association test for this new analysis in the supplemental materials (Figure 4 – Figure Supp. 1).

– A block switch every 10 trials seems very easy to learn (i.e. simply by counting). Did any of the four humans take this strategy?

Thank you for pointing out a lack of clarity in our design description. It is correct that a predictable change would lead to a simple strategy for humans to adopt. In order to avoid this the imposed block switches were generated using a Poisson distribution, so they were stochastic with an average rate ranging from 10 to 30 trials, depending on the testing session.

We can see where this confusion arose, given that we did not have this concern in our simple model (which does not have these sort of anticipatory mechanisms) which we described before the human data. We now clarify the difference in task design between model and human experiments in the Results:

“The experimental task followed the same general structure as our prior work (Bond et al. 2021), with the exception that block switches were deterministic for the model, happening every 10 trials, whereas in actual experiments they are generated probabilistically so as to increase the uncertainty of participant expectations of the timing of outcome switches.”

– I'm a bit puzzled the prediction of CBGT network choices (lines 130-131) is not 100%, since presumably all the relevant information (firing rates of all regions modelled) together should perfectly predict the choice. How crucial is the choice of the LASSO-PCR classifier (over other classifiers)?

Regarding the classifier’s accuracy on the CBGT model, we should point out that this was a probabilistic decision-making task. If the optimal choice is rewarded 75% of the time, we set the other choice as 25% rewarded. This means that selecting the statistically optimal choice 100% of the time would result in an accuracy ceiling of 75%. But, more importantly, this is highlighting what appears to be a bug, but is in fact a feature. Indeed, the fact that the classifier is not at 100% is exactly what we would expect if the network experiences decision uncertainty. This variance is harvested to evaluate our primary hypothesis about uncertainty across action channels and drift rate in the decision process.

As for the second point regarding choice of classifier, the LASSO-PCR approach is a common classifier approach used for building whole brain classifiers (Rasero et al. 2021, Wager et al. 2011). It was specifically designed as a conservative approach to overcoming high model complexity without overfitting and is, by design, an ultra-conservative approach. We could have tried other machine learning approaches for handling high data complexity, but many of these are “black box” approaches that make interpretability more difficult. We could re-run our analysis with ridge regression or elastic net combined with PCR, but these choices are unlikely to change the overall conclusions of the finding because the complete independence of each principal component means that ridge and lasso would converge on largely the same solutions.

But the reviewer is right, that we did not vet the necessity for the double constraint for model complexity. We have run a follow-up analysis on both the model and neuroimaging data without the LASSO regularization step. This largely produced the same accuracy as our model with LASSO penalties. See item 4 for Reviewer #1 under minor concerns for a figure comparing the prediction accuracy and ROC curve for our results with and without regularization.

Given that the choice in classifier parameters does not have a large impact on the results, we have opted to keep the more conservative LASSO-PCR model. We feel that a vetting of different classifier models would shift the focus of the paper to an engineering question (i.e.- maximizing classifier accuracy), rather than the hypothesis-driven focus that drives the current study. However, our data and code are completely publicly available for follow up comparisons of classifier models for predicting human choices. We strongly encourage anyone interested in this follow up question to use these freely.

We now add further justification of our analysis choice to the Methods and the Results, as shown in item 24 of the Minor section for Reviewer #1.

References

Bond, K., Dunovan, K., Porter, A., Rubin, J. E., and Verstynen, T. (2021). Dynamic decision policy reconfiguration under outcome uncertainty. *ELife*, *10*, e65540.

Mink, J. W. (1996). The basal ganglia: focused selection and inhibition of competing motor programs. *Progress in neurobiology*, *50*(4), 381-425.

Rasero, J., Sentis, A. I., Yeh, F. C., and Verstynen, T. (2021). Integrating across neuroimaging modalities boosts prediction accuracy of cognitive ability. *PLoS computational biology*, *17*(3), e1008347.

Verstynen, T., Diedrichsen, J., Albert, N., Aparicio, P., and Ivry, R. B. (2005). Ipsilateral motor cortex activity during unimanual hand movements relates to task complexity. *Journal of neurophysiology*, *93*(3), 1209-1222.

Wager, T. D., Atlas, L. Y., Leotti, L. A., and Rilling, J. K. (2011). Predicting individual differences in placebo analgesia: contributions of brain activity during anticipation and pain experience. *Journal of Neuroscience*, *31*(2), 439-452.

[Editors’ note: what follows is the authors’ response to the second round of review.]

We appreciate your efforts in trying to address the reviewers' points. While the reviewers were satisfied with several of your responses, there are remaining issues. Most importantly, both reviewers felt that the study, while useful, appears a bit preliminary and the evidence in support of your conclusions remains incomplete.

Reviewer #1 (Recommendations for the authors):

The overall responses from the authors were mainly satisfactory, but further concerns remain.

1. Perhaps the authors can replace the spiking neural network model with simpler network-RL-based theoretical models that are more suitably linked and optimised to BOLD-fMRI data or remove the spiking neural network model which can be used and validated in their subsequent future work based on more precise neural recording.

We see why the reviewer thinks a more abstracted model would be appropriate here, given the overall degrees of freedom in the spiking model. The parsimony afforded by more abstracted networks would make the problem *easier*, however, it does not necessarily make the approach more truth-conducive. Rate-based models are largely abstracted and substantially reduce the ability to map to microscale data, as well as capture nuances of the macroscale observations. Had we produced an unreliable result or an inconsistent mapping between the model and empirical data, we could understand the need to “optimize” by moving to a simpler network model. However, here we have a biologically realistic model of a well-described set of pathways that makes an explicit (and accurate) prediction of neuro-behavioral data that we can validate empirically. This removes the need to optimize by moving to a more abstracted model that reduces our ability to map to microscale observations (something we are currently finishing in rodents at the time of this letter). Put another way, would a better match to our empirical data using a rate-based network fundamentally change the conclusions drawn? We do not see how this would. Therefore we have opted to stick with the rate-based network (but see next point).

2. Both CBGT and DDMs' parameters did not seem to be optimised. The authors' justification is that model optimisation based on experimental data could lead to 'circular' inference. Although I can understand this to some extent, especially with physiologically constrained model, I am not sure whether I fully agree with this claim. In any case, the authors should at least summarise clearly which CBGT and DDM parameters were free, which were constrained, and which were tuned – a summary table may help.

We can see why some might come to this conclusion, however, this is not exactly correct. The CBGT network parameters were optimized to produce biologically realistic firing rates. A substantial amount of (unstated) work went into finding algorithms to search the subspace of parameters to produce reasonable physiological and behavioral effects with this network. On the flip side, the DDM parameters were fit to empirical data (both from the network model and human behavior). So they are, by definition, optimized. Note also that in the prior revision we conducted a full model comparison, selecting the model that best explained the observed data according to well-accepted information loss metrics. Free parameters were limited to drift rate and boundary height. The structure of these models is specified in Supplementary Files 1 and 2, both for group-level and participant-by-participant analyses.

Now, it is possible that we just happened to land on a set of CBGT network parameters that produced the hypothesized effects we were searching for. Given the complexity of these sorts of models, this is a reasonable concern. We addressed this in the revision using a simple robustness test of the CBGT network, wherein we varied the parameter schemes within biologically plausible limits and performed the same key analyses as with the previous network configuration. Specifically we chose two more network configurations that produce faster and slower response times (maintaining biological constraints in the firing rates). Overall, we find that the key pattern we observed previously – a reciprocal relationship between drift rate and classifier accuracy – was replicated regardless of parameter scheme. We now include this in the Results section and with supplementary figures (Figure 1 —figure supplements 1 and 2; Figure 2 Figure Supplement 1).

“Next, in order to rule out the possibility that these adaptive network effects emerged due to the specific parameter scheme that we used for the simulations, we re-ran our simulations using different parameter schemes. For this we used a constrained sampling procedure (see Vich et al. 2022) to sample a range of different networks with varying degrees of speed and accuracy. This parameter search was constrained to permit regimes that result in biologically realistic firing rates (Figure 1 —figure supplement 1). The simulations above arose from a parameter scheme lying in the middle of this response speed distribution (Intermediate). We then chose two parameter regimes, one that produces response speeds in the upper quartile of the distribution (Slow) and one that produces response speeds in the lower quartile (Fast; Figure 1 —figure supplement 2A and 2B.). We repeated the simulation experiments with these new more ”extreme” networks. As expected, our general classifier accuracy held across the range of regimes, with comparable performance across all three model types (Figure 1 —figure supplement 2C). In addition, the reciprocal relationship between classifier uncertainty and v were replicated in the Fast and Slow networks (Figure 2 —figure supplement 1A), with the Fast network showing a more expansive dynamic range of drift rates than the Intermediate or Slow networks. When we look at the correlation between classifier uncertainty and v, we again see a consistent negative association across parameter regimes (Figure 2 —figure supplement 1B). The variability of this effect increases when networks have faster response times, suggesting that certain parameter regimes increase overall behavioral variability. Despite this, our key simulation effects appear to be robust to variation in parameter scheme.”

Overall, this work is interesting and could potentially contribute to the computational modelling and neuroscience of adaptive choice behaviour. However, a major component of the work, on the CBGT modelling, seems somewhat premature.

Reviewer #2 (Recommendations for the authors):

I thank the authors for clarifying various technical details.

While this is solid work, I am still unsure as to the main insights we can draw from this paper itself. The main argument for using the fine-scale model to account for fMRI data is that it sets the stage for future work, which will compare these model predictions with mouse data. As much as I understand that this is how science goes, the result for this paper is that linking fMRI and the detailed model is a bit strange (and makes it much harder to draw conclusions from). For the general readership of eLife, I'm not sure if the current insights are very helpful before knowing the results of the more fine-grained data that are currently being collected.

We can see where the reviewer is coming from, however, we respectfully disagree. The computational model makes very specific predictions based on the logic of the biological circuit. This is used in a *generative* framing approach, where the model predicts and data validates (or does not). This approach has been used many times before, including in published work at *eLife*:

Scheinost, D., Noble, S., Horien, C., Greene, A. S., Lake, E. M., Salehi, M., … and Constable, R. T. (2019). Ten simple rules for predictive modeling of individual differences in neuroimaging. Neuroimage, 193, 35-45.

Franklin, N. T., and Frank, M. J. (2015). A cholinergic feedback circuit to regulate striatal population uncertainty and optimize reinforcement learning. *ELife*, 4, e12029.

Schirner, M., McIntosh, A. R., Jirsa, V., Deco, G., and Ritter, P. (2018). Inferring multi-scale neural mechanisms with brain network modelling. *eLife*, 7, e28927.

We now include clarity on this generative modeling approach, and its links to prior work, in the manuscript.

Introduction:

“Here we adopt a generative modeling approach to investigate the underlying neural mechanisms that drive dynamic decision policies in a changing environment. We start with a set of theoretical experiments, using biologically realistic spiking network models, to test how competition within the cortico-basal ganglia-thalamic (CBGT) circuits influences the evidence accumulation process (Dunovan and Verstynen 2019, Bariselli et al. 2018, Mikhael and Bogacz 2016, Rubin et al. 2020, and Yartsev et al. 2018). Our choice of model, over simple abstracted network models (e.g.,rate-based networks), reflects an approach designed to capture both microscale and macroscale dynamics, allowing for the same model to bridge observations across multiple levels of analysis (see also Scheinost et al. 2019, Schirner et al. 2018, and Franklin and Frank 2015).”

Results:

“To simulate this process, we designed a spiking neural network model of the CBGT circuits, shown in Figure 1A, with dopamine-dependent plasticity occurring at the corticostriatal synapses (Rubin et al. 2020, Vich et al. 2020). Critically, although this model simulates dynamics that happen on a microscale, it can be mapped upwards to infer macroscale properties, like inter-region dynamics and complex behavior, making it a useful theoretical tool for bridging across levels of analysis.”

We also add a reference to our approach in the abstract:

“Making adaptive choices in dynamic environments requires flexible decision policies. Previously, we showed how shifts in outcome contingency change the evidence accumulation process that determines decision policies (Bond et al. 2021). Using in silico experiments to generate predictions, here we show how the cortico-basal ganglia-thalamic (CBGT) circuits can feasibly implement shifts in decision policies.”

We also now include a detailed discussion on the limitations of our modeling approach, focusing on critical assumptions of the model that may impact its predictions. This has been added to the Discussion.

“It is important to point out that there are critical assumptions in our model that might impact how the results can be interpreted. For example, we are assuming a strict action channel organization of CBGT pathways (Mink 1996). Realistically action representations in these networks are not as rigid, and there may be overlaps in these representations (see Klaus et al. 2017). However, by restricting our responses to fingers on opposite hands, it is reasonable to assume that the underlying CBGT networks that regulate selecting the two actions are largely independent. Another critical assumption of our model is the simple gating mechanism from the thalamus, where actions get triggered once thalamic firing crosses a specified threshold. In reality, the dynamics of thalamic gating are likely more complicated (Logiaco, Abbott, and Escola 2021) and the nuance of this process could impact network behavior and subsequent predictions. Until the field has a better understanding of the process of gating actions, our simple threshold model, although incomplete, remains useful for generating simple behavioral predictions. These assumptions may limit some of the nuance of the predicted brain-behavior associations, however, they likely have little impact on the main prediction that competition in action representations tracks with the rate of evidence accumulation during decision making.”

Finally, the concern about model complexity is the concern of overparameterization. This would be a significant problem if we were fitting behavioral/neural data to the model. But we are not doing that here. However, to show the robustness of the effects, we now include a broader range of simulations, with different parameter schemes, that show how resilient the predictions are to different network configurations [see reply to Reviewer #1, Comment #2].
